# Regulation of T-cell activation and migration by the kinase TBK1 during neuroinflammation

**DOI:** 10.1038/ncomms7074

**Published:** 2015-01-21

**Authors:** Jiayi Yu, Xiaofei Zhou, Mikyoung Chang, Mako Nakaya, Jae-Hoon Chang, Yichuan Xiao, J. William Lindsey, Stephanie Dorta-Estremera, Wei Cao, Anna Zal, Tomasz Zal, Shao-Cong Sun

**Affiliations:** 1 Department of Immunology, The University of Texas MD Anderson Cancer Center, 7455 Fannin Street, Box 902, Houston, Texas 77030, USA; 2 Division of Pharmacology, School of Medicine, University of Fukui, Fukui 910-8507, Japan; 3 College of Pharmacy, Yeungnam University, Gyeongsan 712-749, Republic of Korea; 4 Department of Neurology, University of Texas Health Science Center at Houston, Houston, Texas 77030, USA; 5 The University of Texas Graduate School of Biomedical Sciences at Houston, Houston, Texas 77030, USA; 6 Center for Inflammation and Cancer, The University of Texas MD Anderson Cancer Center, 7455 Fannin Street, Box 902, Houston, Texas 77030, USA

## Abstract

Development of an immune or autoimmune response involves T-cell activation in lymphoid organs and subsequent migration to peripheral tissues. Here we show that T-cell-specific ablation of the kinase TBK1 promotes T-cell activation but causes retention of effector T cells in the draining lymph node in a neuroinflammatory autoimmunity model, experimental autoimmune encephalomyelitis (EAE). At older ages, the T-cell-conditional TBK1-knockout mice also spontaneously accumulate T cells with activated phenotype. TBK1 controls the activation of AKT and its downstream kinase mTORC1 by a mechanism involving TBK1-stimulated AKT ubiquitination and degradation. The deregulated AKT-mTORC1 signalling in turn contributes to enhanced T-cell activation and impaired effector T-cell egress from draining lymph nodes. Treatment of mice with a small-molecule inhibitor of TBK1 inhibits EAE induction. These results suggest a role for TBK1 in regulating T-cell migration and establish TBK1 as a regulator of the AKT-mTORC1 signalling axis.

## Abstract (web summary)

T cells that are activated by self-antigens in the periphery can migrate into the brain causing neuroinflammatory disease. Here the authors show that TBK1 is necessary for activated T-cell egress from the lymph node, and blocking TBK1 ameliorates autoimmunity in a mouse model of multiple sclerosis.

## Graphical abstract

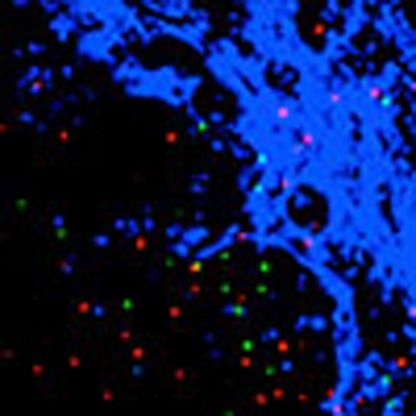

Autoimmunity occurs as a result of T-cell activation by antigens derived from self-tissues^1^. Following priming by the self-antigens in peripheral lymphoid organs, autoimmune effector T cells migrate to target organs to mediate inflammation and tissue damage. The central nervous system (CNS) is an organ of a number of autoimmune and inflammatory disorders, including multiple sclerosis (MS), a disease characterized by chronic inflammation, demyelination and neuronal damage^2^. An animal model, experimental autoimmune encephalomyelitis (EAE), has proved to be powerful for investigating the pathogenesis of MS^3^. It is generally believed that in MS and EAE, autoimmune T cells are primed by myelin-specific antigens and then migrate across the blood–brain barrier to enter the CNS, where they become reactivated and mediate inflammation and neuronal damage^4^,^5^. The T-cell priming and differentiation are governed by signal transduction mediated by the TCR and a costimulatory molecule, CD28, as well as cytokine signals^6^. However, the signalling mechanism that regulates T-cell migration from the lymphoid organs to the tissues of autoimmunity, such as CNS, is still poorly defined.

TBK1, as well as its homologous kinase IKKε, are known as mediators of type I interferon (IFN) induction in antiviral innate immunity^7^,^8^,^9^,^10^,^11^. TBK1 and IKKε share structural homology with IKKα and IKKβ, typical IKK components mediating activation of the transcription factor NF-κB^12^,^13^. However, unlike the typical IKKs, TBK1 and IKKε are dispensable for NF-κB activation but are required for activation of IFN-responsive factor 3, a transcription factor mediating type I IFN gene expression^14^. To date, the roles of the atypical IKKs in other biological processes are poorly defined. In particular, the study of the *in vivo* function of TBK1 has been hampered by the embryonic lethality of the conventional TBK1-knockout (KO) mice^15^.

In the present study, we employed a conditional *Tbk1*-KO approach and demonstrated an unexpected role for TBK1 in the regulation of T-cell function and autoimmunity. T-cell-specific ablation of TBK1 perturbed T-cell homeostasis, characterized by an increased frequency of T cells with an activated phenotype, and rendered naïve T cells more sensitive to activation by agonistic antibodies for TCR and CD28. Surprisingly, the T-cell-conditional *Tbk1*-KO (hereafter called *Tbk1*-TKO) mice were refractory to the induction of EAE due to impaired migration of autoimmune T cells from the draining lymph nodes to the CNS. Our data suggest that TBK1 mediates egress of effector T cells from draining lymph nodes in a mechanism that involves negative regulation of the kinases AKT and mTORC1.

## Results

### TBK1 is a kinase that responds to T-cell activation signals

**Figure 1:**
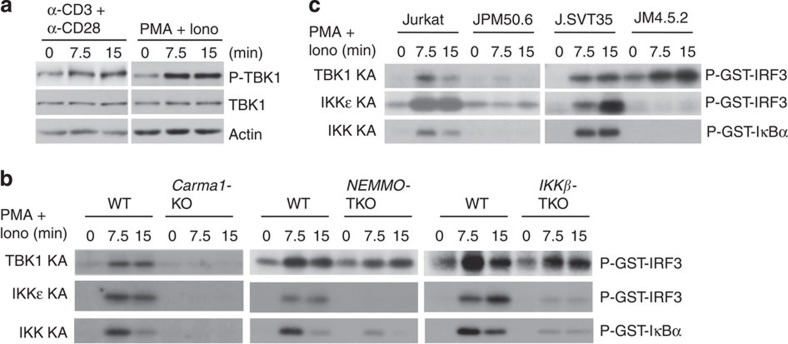
Activation of TBK1 and IKKε by T-cell activation signals. (**a**) IB analysis of phosphorylated (P-) TBK1 (Ser172) and total TBK1 in WT CD4^+^ T cells (6-week-old mice), stimulated with anti-CD3 plus anti-CD28 using a crosslinking method or with the mitogens PMA plus ionomycin. (**b**) CD4^+^ T cells from the indicated KO or T-cell-conditional KO (TKO) and internal WT control mice (6 weeks) were treated with PMA plus ionomycin. TBK1, IKKε and the canonical IKK complex were isolated by IP from the cell lysates and subjected to *in vitro* kinase assays (KA) using GST-IRF3 (for TBK1 and IKKε) or GST-IκBα (for IKK) recombinant proteins as substrates. (**c**) TBK1, IKKε and IKK kinase assays were performed using Jurkat and derivative cells as described in **b**. Data are representative of three or more independent experiments.

Our initial analysis of the BioGPS database revealed that in addition to macrophages, lymphocytes had an abundant expression of TBK1 (data not shown). To assess the function of TBK1 in T cells, we examined its ability to respond to signals stimulated by the TCR and CD28 agonistic antibodies (anti-CD3 and anti-CD28) or mitogens (PMA and ionomycin) that activate protein kinase C and calcium pathways downstream of the TCR and CD28. Despite being known as an innate immune mediator, TBK1, as well as its homologue IKKε, were strongly activated by the T-cell-activation signals, as shown by both phospho-specific immunoblot (IB) and *in vitro* kinase assays (Fig. 1a,b). Activation of the typical IKK complex by T-cell-activation signals requires a scaffold protein, CARMA1 (refs 16, 17). Interestingly, CARMA1 was also required for the activation of TBK1 and IKKε (Fig. 1b). Furthermore, activation of IKKε was completely dependent on IKK, since it was blocked in T cells lacking the IKK regulatory subunit NEMO or the IKK catalytic subunit IKKβ (Fig. 1b). On the other hand, the activation of TBK1 was only partially inhibited in the NEMO- and IKKβ-deficient T cells (Fig. 1b). Similar results were obtained using Jurkat T cells lacking CARMA1 (JPM50.6) (ref. 17) or NEMO (JM4.5.2; ref. 18; Fig. 1c). Thus, both TBK1 and IKKε are activated by T-cell-activation signals, although the underlying mechanism appeared to be different for these kinases.

### TBK1 regulates T-cell activation

**Figure 2:**
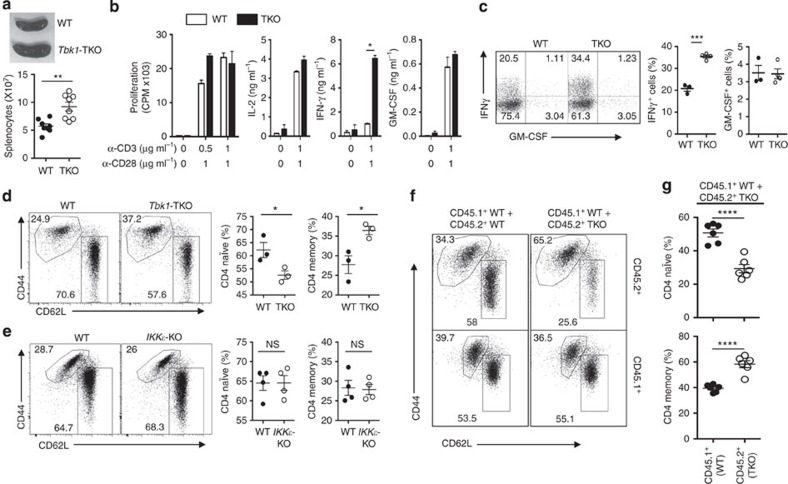
TBK1 deficiency promotes T-cell activation and perturbs T-cell homeostasis. (**a**) A representative spleen picture and mean±s.d. values of spleen cell numbers of 4-month-old WT and *Tbk1*-TKO mice (each circle represents a mouse, *n*=8). (**b**) Proliferation and ELISA (IL-2, IFN-γ and GM-CSF) analyses of CD4^+^ T cells purified from the spleen of young (6 weeks, *n*=4) WT and TKO mice and stimulated for 48 h with the indicated concentrations of plate-bound anti-CD3 and anti-CD28. (**c**) Flow cytometric analyses of splenic CD4^+^ T cells stimulated for 4 h with PMA and ionomycin in the presence of monensin. Frequency of IFN-γ-producing and GM-CSF-producing T cells are presented as a representative fluorescence-activated cell sorting plot (left) and mean±s.d. values (right) of multiple mice (6 weeks, WT=3, TKO=4). (**d**,**e**) Flow cytometric analyses of the frequency of naïve (CD44^lo^CD62L^hi^) and memory (CD44^hi^CD62L^lo^) CD4^+^ T cells in WT and *Tbk1*-TKO (**d**, *n*=3) or WT and *IKKε*-KO (**e**, *n*=4) mice (6 weeks old). Numbers in quadrant indicate the percentage of cells among total CD4^+^ T cells. Data are representative plot (left) and mean±s.d. values (right). (**f**,**g**) Flow cytometric analysis of naïve (CD44^lo^CD62L^hi^) and memory (CD44^hi^CD62L^lo^) CD4^+^ T cells of lethally irradiated *Rag1*-KO mice reconstituted with a mixture (1:1 ratio) of bone marrow cells derived from WT B6.SJL (CD45.1^+^) and WT or TKO (CD45.2^+^) mice (*n*=6). Data are representative plot (**f**) and mean±s.d. values (**g**). Data represent mean±s.d. from three or more independent experiments. *P<0.05; **P<0.01; ***P<0.001; ****P<0.0001; NS, non-significant as determined by two-tailed unpaired Student’s *t*-test, comparing the indicated groups.

To study the role of TBK1 in regulating the T-cell function, we generated *Tbk1*-TKO mice by crossing the *Tbk1*-floxed mice with CD4-Cre mice. As expected, TBK1 was ablated in T cells but not in B cells (Supplementary Fig. 1a). The T-cell-specific TBK1 ablation did not appreciably alter the pattern of thymocyte development (Supplementary Fig. 1b,c). The young (6 weeks old) *Tbk1*-TKO and wild-type (WT) control mice also had comparable numbers of CD4^+^ and CD8^+^ T cells in different lymphoid organs (Supplementary Fig. 1d), suggesting normal survival and homing of naïve T cells in *Tbk1*-TKO mice at young ages. Interestingly, however, at an older age (4 months old), the *Tbk1*-TKO mice had splenomegaly and higher splenocyte numbers (Fig. 2a) as well as increased numbers of CD4^+^ and CD8^+^ T cells in different lymphoid organs and the peripheral blood (Supplementary Fig. 1e).

To examine the effect of TBK1 deficiency on T-cell activation, we used naïve CD4^+^ T cells derived from the 6-week-old mice. When stimulated *in vitro*, the WT and TBK1-deficient naïve CD4^+^ T cells displayed similar level of apoptosis (Supplementary Fig. 1f). Interestingly, however, the TBK1-deficient naïve CD4^+^ T cells displayed a higher ability to proliferate in response to lower doses of anti-CD3 (Fig. 2b). Moreover, the stimulated TBK1-deficient T cells produced remarkably higher levels of IFN-γ, as detected by both enzyme-linked immunosorbent assay (ELISA) and flow cytometry assays (Fig. 2b,c). On the other hand, the TBK1 deficiency did not substantially promote the expression of two other cytokines, interleukin (IL-2) and granulocyte–macrophage colony-stimulating factor (GM-CSF; Fig. 2b,c). Flow cytometry analyses revealed that the Tbk1-TKO mice had a higher frequency of activated or memory-like T cells, characterized by the CD44hiCD62Llo surface markers, and concomitant reduction in the percentage of naïve (CD44loCD62Lhi) T cells (Fig. 2d). This phenotype was not detected in mice lacking the TBK1 homologue, IKKε (Fig. 2e), suggesting a unique function of TBK1. Using a Cre-ER-inducible KO approach, we found that TBK1 ablation in adult mice (Supplementary Fig. 2a) also caused an increase in the frequency of activated T cells in the spleen (Supplementary Fig. 2b) without affecting the thymocyte development (Supplementary Fig. 2c,d). Thus, TBK1 appeared to have a non-redundant role in regulating the steady-state activation and homeostasis of T cells.

Perturbed T-cell homeostasis can be due to a cell-intrinsic mechanism or impaired Treg function. We addressed the mechanism of TBK1 function by mixed bone marrow adoptive transfer. Under such conditions, the WT and *Tbk1*-TKO T cells were developed and maintained in the same environment and in the presence of WT Treg cells, thus allowing the assessment of cell-intrinsic functions of TBK1. We transferred WT bone marrow from WT mice expressing CD45.1 congenital marker (B6.SJL) along with either WT or *Tbk1*-TKO bone marrows (CD45.2^+^) into lethally irradiated *Rag1*-KO mice. As expected, the CD45.1^+^ and CD45.2^+^ WT T cells in the mixed bone marrow chimeric mice had similar frequency of memory and naïve CD4^+^ T cells (Fig. 2f, left panels). In contrast, the *Tbk1*-TKO (CD45.2^+^) CD4^+^ T cells had a profoundly higher frequency of memory population compared with the WT (CD45.1^+^) CD4^+^ T cells within the same chimeric mice (Fig. 2f, right panels, and Fig. 2g). These data suggest that TBK1 has a cell-intrinsic role in the regulation of steady-state activation and homeostasis of T cells.

### TBK1 is essential for EAE induction

**Figure 3:**
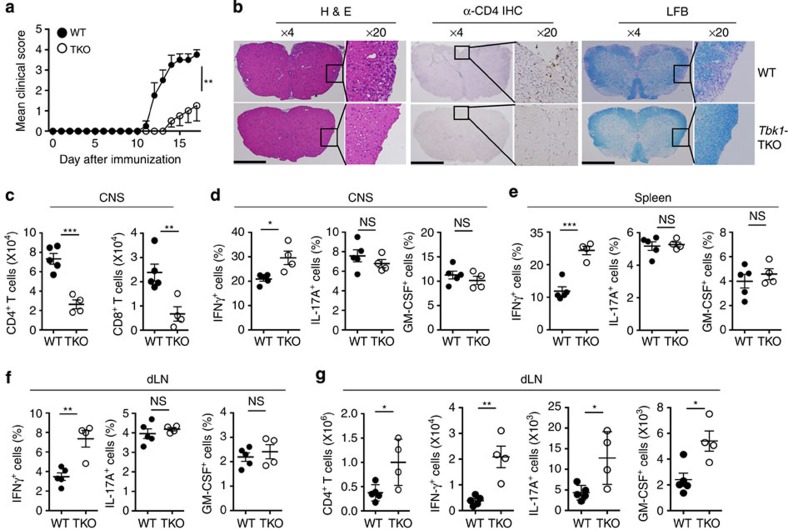
TBK1 is crucial for EAE induction. (**a**) EAE disease score of WT (*n*=5) and *Tbk1*-TKO (*n*=4) mice. (**b**) Haematoxylin and eosin (H&E), CD4 IHC, and Luxol Fast Blue (LFB) staining of spinal cord sections from MOG_35–55_-immunized WT and TKO EAE mice (28 day after immunization). Scale bars, 500 μm. Data are representative of three independent experiments. (**c**) Flow cytometry determining the mean±s.d. values of CD4^+^ T-cell number and CD8^+^ T-cell number recovered from the CNS of WT and *Tbk1*-TKO mice 18 days after EAE induction. (**d**–**f**) Flow cytometric analyses of CD4^+^ T cells from CNS, spleen and lymph nodes of the EAE-induced mice described in **a** (18 days after EAE induction). The T cells were stimulated for 4 h with PMA and ionomycin in the presence of monensin, followed by flow cytometry analysis of the IFN-γ, IL-17A and GM-CSF-producing cells. (**g**) Mean±s.d. values of absolute numbers of IFN-γ, IL-17A- and GM-CSF-producing CD4^+^ T cells recovered from the draining lymph node (dLN) of WT or *Tbk1*-TKO mice described in **a**. Data represent mean±s.d. from three or more independent experiments. *P<0.05; **P<0.01; ***P<0.001; NS, non-significant as determined by two-way analysis of variance with Bonferroni’s post test for EAE clinical scores analysis and two-tailed unpaired Student’s *t*-test for other analysis, comparing the indicated groups.

Since the reduced threshold of T-cell activation often promotes autoimmunity, we examined the role of TBK1 in regulating a T-cell-dependent autoimmunity, EAE. The pathogenesis of EAE involves the inflammatory action of CD4^+^ T helper 17 (Th17) and Th1 cells, which are characterized by the production of the signature cytokines IL-17 and IFN-γ, respectively^3^. Immunization of WT mice with a myelin-specific autoantigen, myelin oligodendrocyte glycoprotein (MOG) peptide MOG_35–55_, led to the induction of overt EAE clinical scores (Fig. 3a). Surprisingly, the *Tbk1*-TKO mice were not hypersensitive, but rather remarkably resistant, to EAE induction (Fig. 3a). Histological analyses revealed drastic reduction in CNS-infiltrating immune cells and CD4^+^ T cells in the *Tbk1*-TKO mice (Fig. 3b, left and middle). In addition, consistent with the clinical scores, the spinal cord of the WT, but not *Tbk1*-TKO, mice had severe demyelination (Fig. 3b, right). Parallel flow cytometry analyses confirmed the drastic reduction in the number of CD4^+^ and CD8^+^ T cells in the CNS of EAE-induced *Tbk1*-TKO mice (Fig. 3c). The EAE-refractory phenotype of the *Tbk1*-TKO mice was unlikely due to a defect in inflammatory T-cell production, since the TBK1-deficient CD4^+^ T cells generated higher levels of Th1 and Th17 cells than WT CD4^+^ T cells in an *in vitro* differentiation assay (Supplementary Fig. 3). Consistently, despite the drastically reduced T-cell numbers in the CNS of *Tbk1*-TKO mice, the percentage of the effector T cells expressing IFN-γ (Th1), IL-17A (Th17) or GM-CSF within the CD4^+^ T-cell population in the CNS, spleen or draining lymph node of *Tbk1*-TKO mice was either higher than or similar to that of the WT control mice (Fig. 3d–f). Notably, the absolute number of the inflammatory effector CD4^+^ T cells was drastically increased in the draining lymph nodes of the *Tbk1*-TKO mice due to the accumulation of total CD4^+^ T cells (Fig. 3g). These results suggest that although TBK1 deficiency promotes T-cell activation and differentiation, it attenuates EAE induction probably by blocking the migration of the effector T cells from the draining lymph nodes to the CNS.

**Figure 4:**
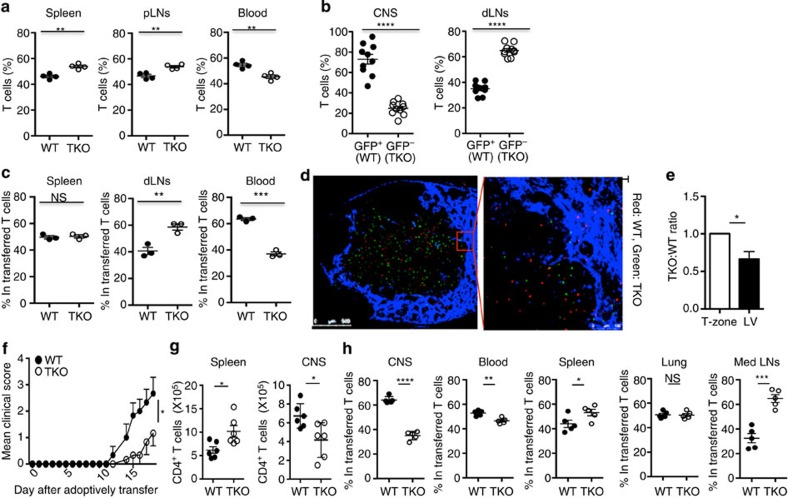
TBK1 deficiency promotes T-cell retention in draining lymph nodes. (**a**) WT and *Tbk1*-TKO naïve CD4^+^ cells were labelled with CMRA and CFSE, respectively, and adoptively transferred into *Tcrb/d*-KO mice in a mixture (at 1:1 ratio). Cell distribution in different organs was analysed 16 h later by flow cytometry (*n*=4). (**b**) Lethally irradiated *Rag1*-KO mice were reconstituted with a mixture (1:1 ratio) of bone marrow cells isolated from *Tbk1*-TKO and GFP-expressing WT mice (*n*=10). The chimeric mice were immunized with MOG_35–55_ 6 weeks after reconstitution. CNS and draining LN (dLN) CD4^+^ T cells were analysed based on GFP expression gated on CD4^+^ cell on day 14 after EAE induction. (**c**–**e**) WT and *Tbk1*-TKO naïve CD4^+^ cell were labelled with CMRA or CFSE respectively and adoptively transferred into WT mice at 1:1 ratio, followed by MOG_35–55_ immunization. Five days later, recipient mice were subjected to flow cytometry analysis of cell distribution in different organs (**c**) or confocal imaging of the differentially labelled T cells in a draining LN, showing the entire T-cell zone (left) and the peripheral region (right) (**d**, red, CMRA-labelled WT T cells; green, CFSE-labelled *Tbk1*-TKO T cells; blue, LYVE-1^+^lymphatic vessels). TKO:WT T-cell ratio in the T-cell zone (set at an index of 1:1) and lymphatic vessel are presented as mean±s.d. values of based on three experiments with a total of >500 T cells analysed (**e**). (**f**,**g**) Clinical scores (**f**) and flow cytometry analysis of CD4^+^ T cells (**g**, day 18) of *Tcrb*/*d*-KO mice undergoing passive EAE induction after being adoptively transferred with WT or *Tbk1*-TKO MOG-primed CD4^+^ T cells. (**h**) Flow cytometry analysis of CD4^+^ T cells in the indicated organs and tissues of WT mice adoptively transferred (for 4 days) with a mixture (1:1 ratio) of MOG-primed WT (CMRA labelled) and *Tbk1*-TKO (CFSE-labelled) CD4^+^ T cells (1:1 ratio). Data represent mean±s.d. from two or more independent experiments. *P<0.05; **P<0.01; ***P<0.001; ****P<0.001; NS, non-significant as determined by two-way analysis of variance with Bonferroni’s post test for EAE clinical scores analysis and two-tailed unpaired Student’s *t*-test for other analysis, comparing the indicated groups.

To further investigate the EAE-regulating function of TBK1, we examined the role of TBK1 in regulating migration of autoimmune T cells to the CNS. Since TBK1 deficiency did not decrease the cellularity or subset composition of T cells in the lymph nodes (Supplementary Fig. 1d,e), it is likely that TBK1 is dispensable for T-cell homing to the lymph nodes. Indeed, adoptively transferred *Tbk1*-TKO CD4^+^ T cells efficiently homed to the spleen and lymph nodes in the T-cell-deficient *Tcrb/d*-KO mice (Fig. 4a). In fact, T-cell cellularity in *Tbk1*-TKO mice was moderately increased in the lymphoid organs and decreased in the peripheral blood, compared with WT mice (Fig. 4a). Thus, although the *Tbk1*-TKO mice displayed reduced frequency of CD62L^hi^ T cells (Fig. 2d), they did not have a defect in T-cell homing to the lymph nodes as shown for the CD62L-deficient mice^19^,^20^.

T-cell migration into the CNS is promoted by the inflammatory CNS microenvironment. To test whether the defect of the *Tbk1*-TKO T cells in CNS infiltration was due to the cell-intrinsic mechanisms, we employed a mixed bone marrow adoptive transfer approach. We adoptively transferred a mixture of green fluorescent protein (GFP^+^) WT bone marrows and GFP^–^ *Tbk1*-TKO bone marrows into lethally irradiated *Rag1*-KO mice and induced EAE in the recipient mice. On the basis of GFP expression, we analysed the frequency of WT and *Tbk1*-TKO T cells in the CNS and draining lymph nodes of the recipient mice during the early effector phase (day 14) of EAE induction. Within the same recipient mice, the WT T cells (GFP^+^) efficiently migrated to the CNS, whereas the majority of the *Tbk1*-TKO T cells (GFP^–^) were retained in the draining lymph nodes (Fig. 4b). Since the WT and TBK1-deficient T cells were activated under the same conditions, these results suggest that the defect of the TBK1-deficient T cells in infiltrating into CNS is not due to attenuated CNS inflammation but rather due to a cell-intrinsic deficiency.

### TBK1 deficiency promotes T-cell retention in lymph nodes

Antigen-activated T cells are initially retained in draining lymph nodes to undergo differentiation and subsequently exit the lymph nodes and migrate to the peripheral site of infection or inflammation^21^. Since the TBK1-deficient T cells were accumulated in the draining lymph nodes during EAE induction (Fig. 4b), we examined whether the loss of TBK1 affected T-cell egress from the lymph nodes, by employing a mixed T-cell transfer approach. We labelled the WT and *Tbk1*-TKO naïve CD4^+^ T cells with CellTracker Orange CMRA dye and carboxyfluorescein succinimidyl ester (CFSE) fluorescence dyes, respectively, and transferred a mixture of the labelled cells (in 1:1 ratio) into WT mice. We then analysed the distribution of the WT and *Tbk1*-TKO T cells during an early phase of MOG immunization (day 5). We detected the accumulation of *Tbk1*-TKO T cells in the draining lymph nodes with concomitantly reduced frequency in the peripheral blood (Fig. 4c). This result indicated enhanced lymph node retention of the TBK1-deficient T cells following antigen stimulation. In parallel, we also performed confocal imaging to analyse the T cells in both the T-cell zone and the lymphatic vessel region. Consistent with the flow cytometry analysis result (Fig. 4c), the CFSE-labelled *Tbk1*-TKO T cells were clearly more abundant than the CMRA-labelled WT T cells in the T-cell zone of the draining lymph node (Fig. 4d, left). Interestingly, however, a significantly lower TKO:WT T-cell ratio was found in the lymphatic vessel area compared with that in T-cell zones (Fig. 4d, right; Fig. 4e), consistent with a reduced egress of TKO T cells through the cortical sinuses (Fig. 4d,e). These results indicate that TBK1 may regulate the exit of effector T cells from the draining lymph nodes.

Since the TBK1 deficiency only partially affected the lymph node exit of T cells, we wondered whether the *Tbk1*-TKO mice were competent in mounting immune responses against infections. In an influenza viral infection model, we found that the lung of the virus-infected *Tbk1*-TKO mice only had moderately reduced frequency of CD4^+^ and CD8^+^ T cells and increased frequency of T cells in the draining mediastinal lymph nodes (Supplementary Fig. 4a). Both the body weight loss (Supplementary Fig. 4b) and the cytokine concentration in bronchoalveolar lavage were comparable between the WT and *Tbk1*-TKO mice (Supplementary Fig. 4c). These results suggest competent immune responses to influenza viral infection in the *Tbk1*-TKO mice.

To understand why the TBK1 deficiency particularly influenced the pathogenesis of EAE, we performed passive EAE studies. We isolated T cells from MOG-immunized WT or *Tbk1*-TKO mice and restimulated them *in vitro* using the MOG peptide. After expansion, we adoptively transferred the autoimmune WT and *Tbk1*-TKO effector T cells intravenously (i.v.) into *Tcrb/d*-KO mice. Interestingly, under these conditions that would bypass the step of T-cell exit from the draining lymph node, the TBK1-deficient T cells were still significantly defective in inducing EAE (Fig. 4f), coupled with reduced CD4 T-cell number in the CNS and increased CD4 T-cell number in the spleen on day 18 following the T-cell transfer (Fig. 4g). Gene expression analysis revealed that the *Tbk1*-TKO T cells had a moderate reduction in the expression of Ninj1 gene and competent expression of several other cell adhesion molecules and chemokine receptors implicated in T-cell migration into the CNS (Supplementary Fig. 5)^22^,^23^,^24^. To further assess the mechanism by which TBK1 mediates CNS infiltration of T cells, we compared the migration of the WT and TBK1-deficient T cells by labelling the *in vitro*-expanded MOG-primed WT and *Tbk1*-TKO T cells with different fluorescence dyes (CMRA and CFSE, respectively) and transferring them in a mixture (1:1 ratio) into WT mice. We then analysed the frequency of the WT and *Tbk1*-TKO T cells (in the same recipients) in the blood, lung and lymphoid organs during the early phase (day 4) of T-cell transfer. This approach further confirmed the defect of the *Tbk1*-TKO effector T cells in migrating to the CNS (Fig. 4h). Interestingly, the *Tbk1*-TKO effector T cells were accumulated in the mediastinal lymph nodes (Fig. 4h). Of note, autoimmune T cells are known to migrate to the CNS via the lung and the lung-draining mediastinal lymph nodes^24^. These results suggest that the impaired CNS infiltration by TBK1-deficient autoimmune T cells may involve increased intranodal retention in different steps, although the involvement of TBK1 in regulating some other aspects of T-cell migration is also possible.

### TBK1 controls activation of AKT and mTORC1

**Figure 5:**
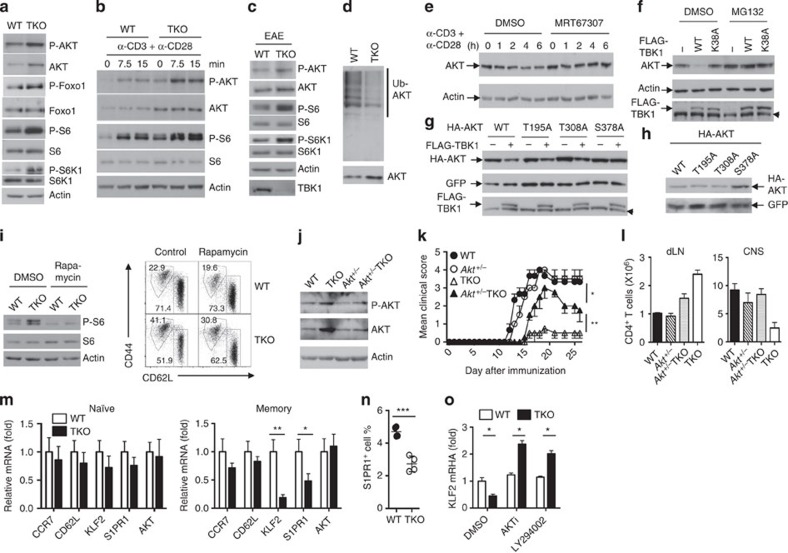
TBK1 stimulates AKT degradation and negatively regulates the activation of AKT and mTORC1 pathways. (**a**) IB analysis of phosphorylated (P-) AKT (Ser473), Foxo1 (Thr24), S6 (Ser235/236), S6K1 (Thr389), as well as their total protein controls in CD4^+^ T cells from WT and *Tbk1*-TKO mice (6 weeks old). (**b**) IB analysis of the indicated phosphorylated (P-) and total proteins in CD4^+^ T cells activated by anti-CD3 plus anti-CD28 using a crosslinking method. (**c**) IB analysis of the indicated phosphorylated (P-) and total proteins in CD4^+^ T cells isolated from day 18 EAE-induced WT or *Tbk1*-TKO mice. (**d**) AKT was isolated by IP from the lysates of thymocytes derived from WT or *Tbk1*-TKO mice (6 weeks old), and the ubiquitinated AKT (Ub-AKT) was detected by IB using an anti-ubiquitin antibody detecting K48-linked polyubiquitin chains. (**e**) IB analysis using WT CD4^+^ T cells, pretreated for 1 h with a TBK1 inhibitor, MRT67307, of solvent control dimethylsulphoxide (DMSO) and subsequently stimulated for the indicated times with anti-CD3 plus anti-CD28 in the presence of a protein synthesis inhibitor, cycloheximide. (**f**) IB analysis of endogenous AKT and Actin and exogenous FLAG-TBK1 in whole-cell lysates of HEK293T cells, transfected as indicated and subsequently incubated for 2 h with DMSO or MG132 (25 μM). An arrowhead indicates the non-specific band. (**g**) IB analysis of the indicated exogenous proteins in the whole-cell lysates of HEK293 cells transfected with HA-AKT WT or the indicated point mutants either in the presence (+) or absence (−) of FLAG-tagged TBK1. An arrowhead indicates the non-specific band. (**h**) IB analysis of whole-cell lysates of WT CD4^+^ T cells transduced with pMIGR1 retroviruses encoding WT or mutant forms of AKT as well as the GFP marker. (**i**) IB (left) and flow cytometry (right) analyses of splenic CD4^+^ T cells from WT or *Tbk1*-TKO mice treated injected (i.p) with rapamycin for 5 days (*n*=5). (**j**) IB analysis of CD4^+^ cells from spleen of the indicated mouse strains. (**k**,**l**) EAE disease score (**k**) and CD4^+^ T-cell number of draining LN and CNS (**l**, day27) (**l**) of the indicated mouse strains, immunized for EAE induction (*n*=4). (**m**) Quantitative PCR (qPCR) analysis of the indicated genes in freshly isolated naïve or memory CD4^+^ T cells from WT and *Tbk1*-TKO mice (*n*=4). (**n**) S1PR1 flow cytometry analysis in memory CD4^+^ cell of inguinal LN from WT or *Tbk1*-TKO mice. (**o**) qPCR analysis of KLF2 in CD4^+^ T cells treated for 1 h with either DMSO control or inhibitors for AKT (AKTi) or PI3 kinase (LY294002). Data represent mean±s.d. from two or more independent experiments. *P<0.05; **P<0.01; NS, non-significant as determined by two-way analysis of variance with Bonferroni’s post test for EAE clinical scores analysis and two-tailed unpaired Student’s *t*-test for other analysis, comparing the indicated groups.

To understand the signalling mechanism by which TBK1 regulates T-cell homeostasis and EAE pathogenesis, we examined the effect of the T-cell-specific TBK1 ablation on the activation of signalling molecules. A striking phenotype of the *Tbk1*-TKO T cells was elevated homeostatic activation of AKT (Fig. 5a). The phosphorylation of Foxo1, a known target of AKT^25^, was also higher in the TBK1-deficient T cells (Fig. 5a). Another major downstream target of AKT is the metabolic kinase mTORC1 (ref. 26). Consistently, the TBK1-deficient T cells had elevated phosphorylation of the mTORC1 substrate protein S6 kinase 1 (S6K1) as well as the S6K1 substrate S6 (Fig. 5a). In contrast, the loss of TBK1 did not alter the phosphorylation of the MAP kinases p38 and ERK (Supplementary Fig. 6a). The crucial role of TBK1 in controlling the homeostatic activation of AKT and mTORC1 was unique, since it was not seen with the IKKε-KO T cells (Supplementary Fig. 6b). Furthermore, the deregulated signalling events in the *Tbk1*-TKO T cells were not due to their activation state, since similar results were obtained with both naïve and memory CD4^+^ T cells (Supplementary Fig. 6c). The loss of TBK1 also promoted the phosphorylation of AKT and S6 stimulated by anti-CD3 plus anti-CD28 (Fig. 5b). Moreover, we detected hyperphosphorylation of AKT as well as S6 and S6K1 in *Tbk1*-TKO T cells isolated during the effector phase of EAE induction (Fig. 5c). Using the Cre-ER-inducible KO system, we found that TBK1 ablation in adult mice also caused aberrant phosphorylation of AKT and S6 in T cells (Supplementary Fig. 6d), further emphasizing a pivotal role for TBK1 in controlling the activation of AKT and its downstream targets, Foxo1 and mTORC1.

### TBK1 induces phosphorylation-dependent AKT degradation

Although AKT activation has been extensively studied, how this master kinase is negatively controlled is unclear. We found that the hyperactivation of AKT in TBK1-deficient T cells was associated with a profound increase in the level of total AKT protein (Fig. 5a and Supplementary Fig. 6b–d). This phenotype was not due to enhanced AKT mRNA expression (Supplementary Fig. 6e), indicating a post-translational mechanism of AKT regulation. We thus examined the possible involvement of the ubiquitin–proteasome pathway. Because of the low number of naïve T cells in the *Tbk1*-TKO mice, we used thymocytes for AKT ubiquitination assays. As seen in peripheral T cells, the TBK1-deficient thymocytes also had elevated level of AKT (Fig. 5d). Interestingly, AKT was undergoing lysine 48-linked polyubiquitination in the WT thymocytes, which was drastically reduced in the TBK1-deficient thymocytes (Fig. 5d). This finding suggests that TBK1 controls the homeostatic level of AKT by promoting ubiquitin-dependent AKT degradation, thus explaining the accumulation of AKT in the TBK1-deficient T cells.

Since the homeostasis of T cells involves constant stimulation of the TCR by self-ligands^27^, we asked whether the TCR signal could stimulate AKT degradation and whether this signalling event was regulated by TBK1. To avoid the homeostatic effect of TBK1 deficiency, we employed WT CD4^+^ T cells. Indeed, the stimulation of the WT CD4^+^ T cells with anti-CD3 and anti-CD28 led to the gradual loss of AKT (Fig. 5e). Moreover, a small-molecule inhibitor of TBK1, MRT67307 (ref. 28), efficiently inhibited the AKT degradation (Fig. 5e). We next employed a transient transfection model to see whether overexpressed TBK1 was able to induce AKT degradation. Indeed, the expression of TBK1, but not its catalytically inactive mutant (K38A), caused drastic loss of endogenous AKT, which was blocked by a proteasome inhibitor, MG132 (Fig. 5f). TBK1 also induced the proteolytic degradation of exogenously expressed AKT but did not affect the co-transfected control protein GFP (Supplementary Fig. 6f).

A previous study suggests that the overexpressed TBK1 phosphorylates AKT at threonine-195 and serine-378, but the functional significance has not been determined^29^. Our finding that TBK1 stimulated AKT degradation led us to examine whether these phosphorylation sites regulate TBK1 stability. Mutation of threonine-195, or the activation-loop phosphorylation site threonine-308, of AKT only weakly inhibited TBK1-stimulated degradation (Fig. 5g). Interestingly, the mutation of serine-378 of AKT completely prevented its degradation by TBK1 (Fig. 5g). When stably expressed in T cells, the AKT mutant harbouring the serine-378 mutation also displayed markedly higher level of expression (Fig. 5h). These results indicate that the TBK1-induced AKT phosphorylation at serine-378 plays an important role in regulating the fate of AKT.

### Functional significance of AKT-mTORC1 deregulation

Our finding that TBK1 controls the activation of AKT and mTORC1 provides molecular insight into the impaired T-cell homeostasis in *Tbk1*-TKO mice, since the AKT-mTORC1 signalling axis has a crucial role in the regulation of T-cell homeostasis^26^. Indeed, we found that treatment of *Tbk1*-TKO mice with an mTORC1 inhibitor, rapamycin, blocked the homeostatic activation of mTORC1 and corrected the T-cell homeostasis phenotype (Fig. 5i).

It is known that activated T cells initially downregulate homing receptors, particularly sphingosine-1-phosphate receptor 1 (S1PR1), and then regain the expression of S1PR1 for migrating out of the lymph nodes^21^. This dynamic change is coupled with the initial activation of AKT and the subsequent decline of the AKT activity. Since *Tbk1*-TKO T cells had aberrant AKT accumulation and activation, we tested whether the ablation of AKT could correct the EAE phenotype of the *Tbk1*-TKO mice. We crossed the *Tbk1*-TKO mice with *Akt*-KO mice to generate *Akt*^+/–^*Tbk1*-TKO mice. Interestingly, ablation of one allele of *Akt*, which reduced the level of AKT and P-AKT in *Tbk1*-TKO T cells (Fig. 5j), largely restored the sensitivity of the *Tbk1*-TKO mice to EAE induction (Fig. 5k) as well as the CNS migration of the *Tbk1*-TKO T cells (Fig. 5l).

A hallmark of the CNS-migrating autoimmune T cells is the high level expression of the transcription factor KLF2 and its target gene S1PR1, the latter of which has become an attractive target for MS therapy^24^,^30^,^31^. In naïve CD4^+^ T cells, the TBK1 deficiency only moderately reduced the expression of KLF2 and S1PR1 as well as two other T-cell homing genes, CCR7 and CD62L (Fig. 5m). Interestingly, however, the TBK1 deficiency greatly inhibited the expression of KLF2 and S1PR1, although only moderately reduced expression of CCR7 and CD62L, in the memory population of CD4^+^ T cells (Fig. 5m). Flow cytometry analyses also revealed reduced level of surface expression of S1PR1 in *Tbk1*-TKO CD4^+^ T cells (Fig. 5n).

The expression of KLF2 is known to be mediated by Foxo1, a transcription factor that is negatively regulated by AKT^32^. Interestingly, incubation of *Tbk1*-TKO CD4^+^ T cells with small-molecule inhibitors for AKT (AKTi) and its upstream regulator PI3 kinase (LY294002) greatly promoted the expression of KLF2 (Fig. 5o). On the other hand, these inhibitors only slightly increased the expression of KLF2 in the WT T cells (Fig. 5o), consistent with the low basal AKT activity in WT T cells (Fig. 5a). Thus, the TBK1 deficiency attenuated the expression of KLF2 and S1PR1, which in turn appeared to be due to the deregulated AKT activation. Collectively, these data indicate that the aberrant AKT activation in TBK1-deficient T cells contributes to their attenuated CNS migration and induction of EAE.

To examine the role of TBK1 in regulating AKT-mTORC1 signalling and gene expression in human T cells, we silenced the expression of TBK1 using short hairpin RNAs (shRNAs) expressed in a lentiviral vector. Interestingly, shRNA-mediated TBK1 silencing promoted the phosphorylation of AKT as well as the AKT-mTORC1 downstream proteins Foxo1 and S6 (Supplementary Fig. 7a). Consistently, the TBK1 knockdown also reduced the expression of S1PR1 and KLF2 as well as CD62L (Supplementary Fig. 7b). Using a well-defined *in vitro* T-cell migration model^33^, we found that TBK1 knockdown in human CD4^+^ T cells significantly inhibited their ability to transmigrate through a human brain microvascular endothelial cell (EC) monolayer (Supplementary Fig. 7c). Similar results were obtained when the T cells were treated with a TBK1 inhibitor, amlexanox (Supplementary Fig. 7d).

In parallel with the functional studies, we analysed the potential alterations of TBK1 expression in MS patients. Our data revealed that the expression of TBK1 is significantly increased in the peripheral blood mononuclear cell (PBMC) of MS patients compared with the healthy donors (Supplementary Fig. 7e). Consistently, TBK1 expression was also shown to be elevated (2.41- and 1.79-folds) in two MS PBMC microarray databases^34^,^35^. These results suggest that TBK1 controls the AKT-mTORC1 signalling axis in both murine and human T cells.

### A TBK1 inhibitor ameliorates EAE pathogenesis

**Figure 6:**
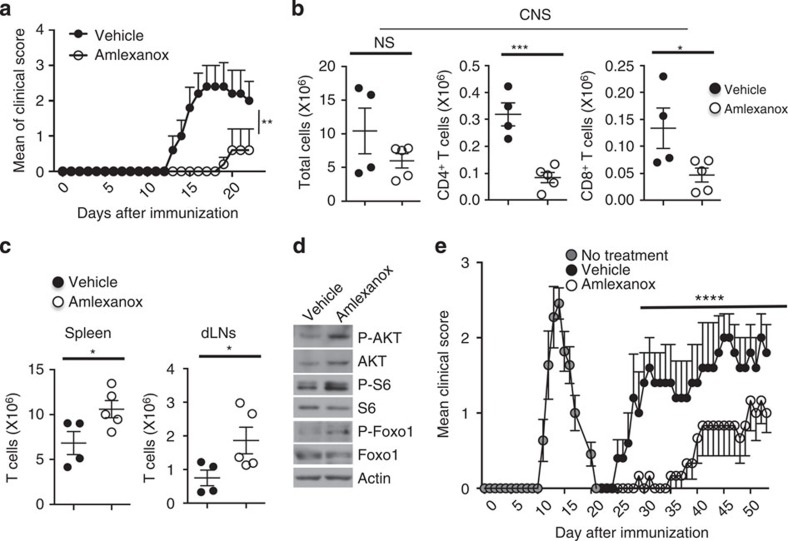
A small-molecule inhibitor of TBK1 promotes AKT-mTORC1 activation and inhibits EAE induction. (**a**–**d**) WT mice were immunized for EAE induction. Starting from 4 days before the immunization, the mice were treated daily (via i.p. injection) with vehicle control or the TBK1 inhibitor amlexanox (25 mg per kg body weight) for 14 days (*n*=5). The immunized mice were monitored for EAE disease scores (**a**) or killed on day 22 post immunization for flow cytometric analysis of the indicated cell populations in the CNS (**b**) and peripheral lymphoid organs (**c**) or for IB analysis of the indicated phosphorylated (P-) and total proteins in the splenic CD4^+^ T cells (**d**). (**e**) EAE clinical score of SJL.J mice immunized with PLP_139–151_ to induce the remitting–relapsing EAE and then treated daily (via i.p. injection) with vehicle control or the TBK1 inhibitor amlexanox (25 mg per kg body weight) for 14 days starting from day 23 (*n*=5). Data represent mean±s.d. from two or more independent experiments. *P<0.05; **P<0.01; ***P<0.001; ****P<0.0001; NS, non-significant as determined by two-way analysis of variance with Bonferroni’s post test for EAE clinical scores analysis and two-tailed unpaired Student’s *t*-test for other analysis, comparing the indicated groups.

The data described above not only revealed a previously unknown signalling mechanism regulating T-cell function and CNS inflammation but also implicated TBK1 as an attractive therapeutic target for the treatment of MS. To further assess the therapeutic value of TBK1, we tested the effect of TBK1 pharmacological inhibition on the induction of EAE. In this regard, a recent study identified the Food and Drug Administration-approved therapeutic compound amlexanox as a selective inhibitor of TBK1 (ref. 36). We asked whether amlexanox could ameliorate EAE and, if so, whether it acted through modulating T-cell migration into the CNS. We treated the mice daily with amlexanox (via intraperitoneal (i.p.) injection) during the induction of EAE. The amlexanox treatment drastically delayed the onset, and reduced the severity, of the EAE disease (Fig. 6a), which was associated with a profound reduction in the number of CD4^+^ and CD8^+^ T cells in the CNS of the EAE-induced mice (Fig. 6b). As seen with the TBK1 gene ablation, the amlexanox treatment did not inhibit T-cell expansion but rather caused the sequestration of T cells in the draining lymph nodes and spleen (Fig. 6c). Moreover, the T cells isolated from the amlexanox-treated mice displayed aberrant activation of AKT and its target protein Foxo1 as well as the mTOCR1 pathway component S6 (Fig. 6d).

To further assess the therapeutic potential of amlexanox, we employed a remitting–relapsing EAE model, which involved the immunization of SJL mice with a peptide (139–151) derived from the proteolipid protein (PLP). As expected, the immunized SJL mice developed EAE, recovered from the disease after about 20 days and then relapsed (Fig. 6e). On day 23 after immunization, we treated the mice (via i.p. injection) with vehicle control or the TBK1 inhibitor amlexanox (25 mg per kg body weight) for 14 days (*n*=5). Importantly, the amlexanox treatment greatly inhibited the EAE relapse (Fig. 6e). Collectively, these results highlight the potential of TBK1 as a therapeutic target for the treatment of MS. Of course, it is currently unclear whether the effect of amlexanox was solely on T cells or also on innate immune cells, such as monocytes or macrophages. Future studies will further address this question by generating myeloid cell-conditional *Tbk1*-KO mice.

## Discussion

The data presented in this study establish TBK1 as a crucial signalling factor that regulates the homeostasis and inflammatory function of T cells. A prominent phenotype of the *Tbk1*-TKO mice was the increased frequency of memory-like T cells and decreased frequency of naïve T cells in the lymphoid organs. Although the TBK1 deficiency promotes T-cell activation and differentiation, the *Tbk1*-TKO mice are refractory to the induction of EAE, an animal model of the autoimmune neuroinflammatory disease MS.

We obtained genetic and biochemical evidences that TBK1 served as a negative regulator of AKT. T-cell-specific ablation of TBK1 caused heightened activation of AKT and its downstream targets, mTORC1 and Foxo1. The deregulated mTORC1 activation appeared to contribute to the perturbed homeostasis of TBK1-deficient T cells. We found that the injection of *Tbk1*-TKO mice with an mTORC1 inhibitor could largely, although not completely, correct their abnormal phenotype in T-cell homeostasis. These data suggest that the uncontrolled mTORC1 signalling contributes to, but may not be solely responsible for, the perturbed T-cell homeostasis in *Tbk1*-TKO mice. Besides mTORC1, several other factors in the related pathways, including the transcription factors Foxo1 and KLF2, are required for maintaining T-cell homeostasis^37^,^38^. Indeed, the TBK1 deficiency attenuated Foxo1 activation and KLF2 expression in T cells, indicating the involvement of these transcription factors in the perturbed T-cell homeostasis in the *Tbk1*-TKO mice.

Migration of autoimmune T cells from lymphoid organs to the target tissues represents a crucial and complex step in the development of autoimmunity. Newly activated T cells express low levels of the factors involved in T-cell migration, particularly the transcription factor KLF2 and its target gene product S1PR1, which is correlated with the lymphoid retention of the T cells^39^. Over time, the activated T cells differentiate into effector T cells and gradually regain the ability to express the migration-regulatory genes, which is important for the egress of T cells from the lymphoid organs. The role of KLF2 and S1PR1 in mediating T-cell migration has been directly demonstrated in a number of studies^37^,^40^,^41^. Our data suggest that TBK1 promotes the expression of KLF2 and S1PR1 in T cells and, thereby, facilitates the lymph node egress of T cells. We obtained genetic and pharmacological evidence that deregulated AKT activation in *Tbk1*-TKO T cells contributed to the reduced expression of KLF2 and S1PR1 and attenuated migration of T cells to the CNS. Ablation of one allele of *Akt* in the *Tbk1*-TKO largely corrected their phenotype in T-cell migration and EAE sensitivity. The transcription factor Foxo1, which is negatively regulated by AKT, has been implicated in the induction of KLF2 and other migration-regulatory genes^38^,^42^,^43^. We propose that TBK1 promotes T-cell migration by downregulating AKT and, thereby, maintaining a sufficient level of active Foxo1 for mediating the expression of migration genes. Indeed, we found that TBK1 deficiency causes elevated Foxo1 phosphorylation in T cells.

It is important to note that the TBK1 deficiency only partially impaired the lymph node egress of T cells. Consistently, the *Tbk1*-TKO mice remained competent in mounting immune responses to influenza virus infection. This finding suggests that TBK1 may regulate additional functions of the CNS autoimmune T cells other than facilitating their exit from the draining lymph nodes. It is also possible that the complex route of autoimmune T-cell migration in EAE may contribute to the remarkable resistance of the *Tbk1*-TKO mice to EAE induction. Following their exit from draining lymph nodes, CNS autoimmune T cells migrate to the CNS via the lung and the lung-draining mediastinal lymph nodes^24^. Our data suggest that the *Tbk1*-TKO effector T cells not only have the tendency to be retained in the draining lymph nodes but also accumulate in the mediastinal lymph nodes. These results suggest that the impaired CNS infiltration of TBK1-deficient T cells may involve different steps of lymph node retention, although it remains possible that TBK1 may also regulate additional mechanisms of T-cell migration to the CNS.

TBK1 and its homologue IKKε are known as an innate immune kinase that mediates induction of type I IFNs^14^. Although TBK1 is thought to be functionally redundant with IKKε in innate immune cells, our data suggest that these two kinases have fundamentally different functions in T cells. While TBK1 regulates T-cell homeostasis and mediates T-cell migration into the CNS, IKKε is dispensable for these biological functions. IKKε is also dispensable for the control of AKT-mTORC1 signalling axis. Nevertheless, like TBK1, IKKε is activated by the T-cell activation signals. Further studies are required to examine whether IKKε regulates any aspects of T-cell functions. The functional differences between TBK1 and IKKε have also been shown in B cells, in which TBK1, but not IKKε, regulates antibody class switching to IgA^44^.

Studies using immortalized mouse embryonic fibroblast and cancer cell lines suggest a positive role of TBK1 in the regulation of AKT^29^,^45^. TBK1 phosphorylates AKT at several residues, including threonine-195 and serine-378, although the functional significance has not been defined^29^. Our current work provides the first evidence for the *in vivo* role of TBK1 in the regulation of AKT. Our data clearly demonstrated a negative role for TBK1 in AKT regulation. TBK1 does not seem to inhibit the catalytic activation, but rather controls the fate, of AKT. In TBK1-deficient T cells, the steady level of AKT is drastically elevated, which contributes to the aberrant AKT activity in naïve and effector T cells. We have obtained biochemical evidence that the serine-378 phosphorylation site of AKT is crucial for TBK1-stimulated AKT degradation.

Our work not only demonstrated a novel signalling mechanism that regulates T-cell homeostasis and EAE pathogenesis but also have profound implications for MS therapy. Regarding the human relevance of our findings, we found that TBK1 knockdown in human PBMCs causes hyperactivation of AKT-mTORC1 signalling and reduced expression of the T-cell homing genes KFL2 and S1PR1. Furthermore, TBK1 knockdown or pharmacological inhibition attenuates the transmigration of human CD4^+^ T cells across a human brain microvascular endothelial monolayer *in vitro*. Our finding that TBK1 ablation does not affect T-cell activation or immune responses against flu viral infection, but attenuates EAE induction, makes TBK1 an attractive therapeutic target to be exploited in the treatment of MS. We found that amalexanox, a Food and Drug Administration-approved drug that selectively inhibits TBK1 (ref. 36), greatly attenuated the induction of EAE and inhibited the disease relapse in a remitting–relapsing model of EAE. Amlexanox also inhibited human T-cell migration *in vitro*. Of note, the mode of action of TBK1 differs substantially from that of S1PR1. First of all, TBK1 appears to regulate the re-expression of S1PR1 following its downregulation in activated T cells, but is dispensable for the overall expression of S1PR1. Thus, unlike the S1PR1 ablation, the TBK1 deficiency does not influence the homing of naïve T cells or the thymic emigration of newly generated T cells. Furthermore, the TBK1 deficiency only partially inhibits the expression of S1PR1 and the egress of effector T cells from the lymph nodes. Consistently, the loss of TBK1 does not appreciably compromise immune responses against influenza virus infection. These findings along with the fact that amlexanox is a relatively safe drug that has been used for many years in the clinic indicate that amlexanox may be an attractive compound to be evaluated in MS clinical trials.

## Methods

### Mice

The *Tbk1*-flox mice were generated in B6 × 129 mixed genetic backgrounds^44^ and further backcrossed to the B6 mice for four generations. To generate T-cell-conditional *Tbk1*-KO mice, *Tbk1*-flox mice^44^ were crossed with CD4-Cre transgenic mice (B6 genetic background, Jackson Laboratories), producing *Tbk1*^+/+^CD4-Cre (named WT) and *Tbk1*^fl/fl^CD4-Cre (named *Tbk1*-TKO mice) mice. *Tbk1*-flox mice were also crossed with Rosa26-Cre-ER mice (B6 genetic background, Jackson Laboratories) and the progeny *Tbk1*^+/+^Rosa26-Cre-ER and *Tbk1*^fl/fl^Rosa26-Cre-ER mice were injected i.p. with tamoxifen (2 mg per mouse) in corn oil daily for four consecutive days to induce Cre function. The tamoxifen-treated *Tbk1*^+/+^Rosa26-Cre-ER and *Tbk1*^fl/fl^Rosa26-Cre-ER mice were named WT-ER and *Tbk1*-ER mice, respectively, and used 2 weeks after the treatment. *Carma1*-KO mice (B6 × 129 genetic background) were provided by Dr Josef M. Penninger (Austrian Academy of Sciences)^46^. Mice with loxP-flanked allele encoding IKKβ (IKK2) or NEMO alleles were provided by Dr Manolis Pasparakis (University of Cologne)^47^. These mice were crossed with CD4-Cre mice to generate the T-cell-conditional IKKβ KO (*IKKβ*-TKO) and NEMO KO (*NEMO*-TKO) mice. *IKKε*-KO, B6.SJL (CD45.1^+^), C57BL/6, *Rag1*-KO and *TCRβ*/*δ* KO mice were from Jackson Laboratory. Experiments were performed with age-matched mice. Mice were maintained in a specific pathogen-free facility of The University of Texas MD Anderson Cancer Center, and all animal experiments were done in accordance with protocols approved by the Institutional Animal Care and Use Committee of the University of Texas MD Anderson Cancer Center.

### Cell lines

The NEMO-deficient JM4.5.2 cell line and its NEMO-competent control J.SVT35 are Jurkat derivatives^18^. The CARMA1-deficient JPM50.6 mutant cell line and its parental Jurkat cell line were provided by Dr Xin Lin (UT MD Anderson Cancer Center)^17^.

### Plasmids and antibodies and reagents

The pcDNA expression vector encoding haemagglutinin (HA)-tagged human AKT1 was provided by Dr Keqiang Ye (Emory University). pMIGR1-hAKT was generated by inserting human AKT1 cDNA into the EcoRI and BglII sites of pMIGR1 vector, and the AKT mutants (T195A, T 308A, S378A) were created by site-directed mutagenesis. Flag-tagged TBK1 and its catalytically inactive mutant, K38A, were provided by Dr Chen Wang (Shanghai Institutes for Biological Sciences). GST-IκBα (amino acids 1–54 of IκBα) was as described^48^ and GST-IRF3 (amino acids 380–427) was provided by Dr Rongtuan Lin (McGill University).

Functional grade anti-mouse (m) CD3ε (145-2C11) and anti-mCD28 (37.51) antibodies and blocking antibodies for mIFN-γ (XMG1.2) and mIL-4 (11B11) were from eBioscience. Antibodies for AKT1 (B-1, 1:1,000), phospho-ERK1/2 (E-4, 1:1,000), ERK1/2 (K-23, 1:1,000), p38 (H-147, 1:1,000), and GFP (B-2, 1:1,000) were from Santa Cruz Biotechnology. Anti-IKKi (IKKε, 1:1,000), anti-Actin (C-4, 1:5,000) and horseradish peroxidase-conjugated anti-Flag (M2, 1:10,000) were from Sigma-Aldrich. Antibodies for phospho-TBK1 Ser172 (D52C2, 1:1,000), phospho-AKT1 Ser473 (D9E, 1:1,000), phospho-S6K1 Thr389 (1A5, 1:1,000), phospho-S6 Ser235/236 (D57.2.2E, 1:1,000), phospho-FoxO1 Thr24/FoxO3a Thr32 (1:1,000), phospho-p38 Thr180/Tyr182 (3D7, 1:1,000), TBK1, S6K1 (49D7, 1:1,000), S6 (54D2, 1:1,000) and FoxO1 (C29H4, 1:1,000) were from Cell Signaling Technology. Horseradish peroxidase-conjugated anti-haemagglutinin (HA-7, 1:3,000) was from Roche. Anti-K48-Ubiquitin (Apu2, 1:1,000) was from Millipore.

Fluorescence-labelled antibodies for murine (m) CD4 (L3T4, 1:300), mCD8 (53-6.7, 1:300), mCD3 (145-2C11, 1:300), CD44 (IM7, 1:300), CD62L (MEL-14, 1:300), mTCRβ (H57-597, 1:300), mCD45.1 (A20, 1:300), mCD45.2 (104, 1:300), mThy1.1 (HIS51, 1:300), IL-17A (eBio17B7, 1:300), and IFN-γ (XMG1.2, 1:300) were purchased from eBioscience. Annexin V was from BD. The fluorescence-labelled phospho-specific antibodies were all from Cell Signaling Technology.

The AKT inhibitor 1/2 was from Calbiochem, the PI3 kinase inhibitor LY294002 was from Selleck, the TBK1 inhibitor MRT673037 was from Chemexpress, the TBK1 inhibitor amlexanox was from Cayman Chemical and the protein synthesis inhibitor cycloheximide was from Sigma-Aldrich.

### Flow cytometry analysis and cell sorting

Single-cell suspensions of splenocytes and lymph node cells were subjected to flow cytometry and cell sorting as previously described^49^ using a FACSAria (BD Biosciences). For intracellular cytokine staining (ICS) assays, T cells isolated from the spleen, draining lymph nodes or CNS (brain and spinal cord) of immunized mice or from *in vitro* cultures were stimulated for 4 h with PMA (50 ng ml^−1^) and Ionomycin (500 ng ml^−1^) in the presence of monensin (10 μg ml^−1^). The stimulated cells were fixed in 2% paraformaldehyde and permeablized in 0.5% saponin and then subjected to cytokine staining flow cytometry analyses.

### *In vivo* inhibition of mTORC1

Mice were injected daily (i.p) with the mTORC1 inhibitor rapamycin (3 mg per kg body weight) or solvent control (1% ethanol in PBS) for 5 days and then killed for experiments.

### Influenza viral infection

Influenza virus A/PR8 (H1N1) was provided by Dr Brian E. Gilbert (Baylor College of Medicine). For influenza infection, mice were first anaesthetized and then inoculated intranasally with 50 μl of influenza virus A/PR8 diluted in DMEM medium. Control mice were inoculated with 50 μl DMEM as control. Body weight was monitored every other day for 8 days. On day 8, bronchoalveolar lavage fluid was collected in anaesthetized mice. Then, 500 μl cold PBS was instilled into the lungs through the trachea and aspirated back. After collecting bronchoalveolar lavage fluid, mice were killed for cell distribution analysis.

### Induction and evaluation of EAE

The encephalitogenic peptide of MOG (residues 35–55, Met-Glu-Val-Gly-Trp-Tyr-Arg-Ser-Pro-Phe-Ser-Arg-Val-Val-His-Leu-Tyr-Arg-Asn-Gly-Lys) and the encephalitogenic peptide of myelin PLP (residues 139–151, His-Cys-Leu-Gly-Lys-Trp-Leu-His-Pro-Asp-Lys-Phe) were purchased from Genemed Synthesis Inc. (San Francisco, CA, 95% purity). To induce acute EAE, mice were injected subcutaneously (in the back region) with 200 μg of the MOG_35–55_ peptide in CFA containing 5 mg ml^−1^ heat-killed *Mycobacterium tuberculosis* (H37Ra strain; BD Diagnostics, Franklin Lakes, NJ). To induce relapse EAE, SJL.J mice were immunized with 150 μg of PLP_139–151_ in CFA containing 5 mg ml^−1^ heat-killed *Mycobacterium tuberculosis*. For both types of induction, on the day of immunization and 48 h later, the mice were also injected i.v. with Pertussis toxin (200 ng per mouse; List Biological Laboratories, Campbell, CA) in PBS. Mice were examined daily for EAE disease symptoms, which were scored using a standard method: 0, no clinical signs; 1, limp tail; 2, paraparesis (weakness, incomplete paralysis of one or two hind limbs); 3, paraplegia (complete paralysis of two hind limbs); 4, paraplegia with fore limb weakness or paralysis; and 5, moribund state or death. The EAE scores were assessed without group allocation information.

For amlexanox treatment, mice were i.p. injected with amlexanox (25 mg per kg body weight) or vehicle (dimethylsulphoxide 50% and corn oil 50%) control daily for total of 14 days. Mice were randomly selected for receiving amlexanox treatment.

For passive EAE, WT and *Tbk1*-TKO mice were immunized with MOG_35–55_ peptide for 10 days. Draining LN and spleen cells were then isolated and *in vitro* expanded for 72 h in the presence of 30 μg ml^−1^ MOG_35–55_ peptide and Th17-differentiation agents (10 μg ml^−1^ anti-IFNγ, 10 ng ml^−1^ IL-1β and 10 ng ml^−1^ IL-23). CD4^+^ T cells were purified and adoptively transferred into irradiated (500 rads) recipient mice to for passive EAE induction.

### Analysis of *in vivo* T-cell differentiation and CNS infiltration

At the indicated times after MOG_35–55_ immunization, the mice were killed for splenocyte preparation and CNS infiltration analysis. CD4^+^ T cells were isolated from the splenocytes using magnetic beads (Invitrogen) and subjected to ICS as described above. For the preparation of CNS lymphocytes, brains and spinal cords were excised and dissociated for 1 h at 37 °C by digestion with collagenase IV (0.5 mg ml^−1^; Invitrogen) and DNase I (10 μg ml^−1^; Roche, Indianapolis, IN) in RPMI medium. Dispersed cells were passed through a 40-μm nylon mesh and collected by centrifugation. The cells were then resuspended in RPMI medium, layered onto a Percoll density gradient (Biochrom, Berlin, Germany) and centrifuged for 30 min (625 *g*, 22 °C). CNS lymphocytes were isolated by collection of the interphase fraction between 30 and 70% Percoll. After intensive washing in Hanks balanced-salt solution, cells were analysed by flow cytometry.

### Histology and immunohistochemistry

We dissected spinal cords from mice transcardially perfused with 4% (w/v) paraformaldehyde and postfixed them overnight. We then stained the paraffin-embedded sections (8 μm) of spinal cords with haematoxylin and eosin or Luxol Fast Blue staining. Cryostat sections (7–8 μm) of lymph nodes were fixed and stained with biotinylated anti-CD4 (1:100; BD Biosciences) at 4 °C overnight, followed by HRP-conjugated streptavidin (Thermo Scientific) at room temperature for 1 h. Slices were then developed with peroxidase substrate kit (Vector).

### Mixed bone marrow chimera

*Rag1*-KO mice were subjected to lethal-dose (992 rads) irradiation and, 1 day later, were adoptively transferred with bone marrow cells harvested from the tibiae and femurs of the indicated mice. For mixed bone marrow transfers, an equal number (6.4 × 10^6^) of bone marrows from the CD45.1^+^ B6.SJL and the CD45.2^+^ WT or *Tbk1*-TKO mice or from the GFP^+^ WT and the GFP^–^ *Tbk1*-TKO mice were mixed and injected i.v. into the irradiated *Rag1*-KO mice. After 6 weeks, the resulting chimeras were either directly analysed or subjected to EAE induction.

### Adoptive transfer of T cells for homing studies

WT and *Tbk1*-TKO T cells were incubated at 37 °C for 15 min with 1 mM CFSE and 5 mM CMRA (Molecular Probes), respectively, according to manufacturer’s protocol. The differentially labelled WT and *Tbk1*-TKO T-cell preparations were mixed at 1:1 ratio (5 × 10^6^ cells each) and injected i.v. into the indicated recipient mice, followed by immunizing the mice with MOG_35–55_ peptide. Lymphatic vessels and the draining inguinal lymph nodes were labelled *in vivo* by injecting mice (subcutaneous) with 5 μg of anti-mouse LYVE-1-AF647 antibody in 100 μl PBS in the flank 24 h before imaging. For static imaging, inguinal LNs were excised, fixed in 3% paraformaldehyde PBS and cut in half. Each half lymph node was whole-mounted on a cover slip imaged using Leica SP5 RS AOBS confocal microscope with a × 20 NA0.75 objective and silicon immersion. Three-dimensional image stacks were acquired with 4 μm *z*-spacing. Three-dimensional analysis was performed using Imaris 7.7 software.

### T-cell purification and *in vitro* treatments

CD4^+^ T cells were purified from splenocytes with anti-CD4-conjugated magnetic beads (Invitrogen). Inhibitor studies were carried out by culturing the cells in 24-well plates (4 × 10^6^ cells per well) in the presence of AKT1/2 inhibitor (AKTi, 1 μM), the PI3 kinase inhibitor (LY294002, 50 μM) or the TBK1 inhibitor (MRT67307, 2 μM). For ELISA and proliferation assays, naïve CD4 T cells were stimulated in replicate wells of 96-well plates (1 × 10^5^ cells per well). Culture supernatants were analysed by ELISA (eBioScience), and cells were pulse-labelled for 6 h with [^3^H]thymidine for proliferation assays.

For *in vitro* CD4^+^ T-cell differentiation assays, naïve CD4^+^ T cells (CD4^+^CD25^−^CD44^lo^CD62L^hi^) were sorted from splenic CD4^+^ T cells, prepared using a CD4 T-cell Isolation Kit (Miltenyi Biotec, Auburn, CA) and stimulated with plate-bound anti-CD3 and anti-CD28 under Th0 (5 μg ml^−1^ anti-IL-4, 5 μg ml^−1^ anti-IFN-γ), Th1 (10 ng ml^−1^ IFN-γ, 10 ng ml^−1^ IL-12, 5 μg ml^−1^ anti-IL-4) or Th17 (20 ng ml^−1^ IL-6, 5 ng ml^−1^ TGF-β, 5 μg ml^−1^ anti-IL-4, 5 μg ml^−1^ anti-IFN-γ) conditions. After the indicated times, the cells were subjected to ICS to quantify the production of their signature cytokines.

### Retroviral and lentiviral infections

Retroviral particles were prepared using the indicated pMIGR1-GFP-based expression vectors, as previously described^50^. For the production of lentiviral particles, HEK293T cells were transfected (by calcium method) with pLKO.1 lentiviral vectors encoding shRNAs for either the control luciferase protein or TBK1 along with the packaging vectors psPAX2 and pMD2. Naive CD4^+^ T cells were activated with plate-bound anti-CD3 (1 μg ml^−1^) plus anti-CD28 (1 μg ml^−1^) in 24-well plates for 36 h and then infected with retroviruses. After 48 h, the transduced cells were either enriched by flow cytometric cell sorting (based on GFP expression) for *in vitro* assays or directly transferred into T-cell-deficient mice for *in vivo* assays.

PBMCs were isolated from human whole blood samples (purchased from The Gulf Coast Regional Blood Center) by Ficoll density gradient centrifugation. The cells were activated for 24 h with anti-CD3 plus anti-CD28 and then infected with the control or TBK shRNA lentiviral particles. Following infection, the infected cells were selected by culturing the cells in the presence of puromycin for 48 h. The use of human blood samples was approved by the Institutional Review Board for Human Research at MD Anderson Cancer Center.

### *In vitro* human T-cell migration assays

*In vitro* T-cell migration assays were performed using a 24-well plate Boyden chamber essentially as previously described^33^. We seeded 1 × 10^5^ primary human brain microvascular ECs (ACBRI 376) on top of a 3-μm pore size membrane in endothelial cell culture media. After 5 days of cultivation, the cells formed a confluent monolayer. At that point, the ECs were treated for 24 h with 100 ng ml^−1^ of recombinant human IL-17 (R&D Systems). The next day, ECs were washed and a suspension of 1 × 10^6^ CD4^+^ human T cells was loaded in the upper chamber. The migration ability of T cells was assessed by counting the absolute number of T cells that transmigrated to the lower chamber after 18 h.

### Immunoblot and kinase assays

For protein phosphorylation analysis and kinase assays, purified T cells were stimulated by a crosslinking method^49^. In brief, the cells were incubated on ice with anti-CD3 (2 μg ml^−1^) and anti-CD28 (2 μg ml^−1^), followed by crosslinking with goat anti-hamster Ig (25 μg ml^−1^) for different time periods at 37 °C and then immediately lysed in a kinase cell lysis buffer supplemented with phosphatase inhibitors. For IB analyses using transiently transfected cells, HEK293T cells were transfected using calcium method and cultured for 48 h. The cells were either treated as indicated or directly collected for lysate preparation. IB analyses were performed using antibodies that detect phosphorylated AKT (Ser473, 1:1,000), Foxo1 (Thr24, 1:1,000), S6 (Ser235/236, 1:1,000), S6K1 (Thr389, 1:1,000), p38 (Thr180/Tyr182, 1:1,000) and ERK1/2 (Tyr204, 1:1,000), or their total protein controls. *In vitro* kinase assays were as described^51^.

For ubiquitination assays, thymocytes were isolated from young (6 weeks old) WT or *Tbk1*-TKO mice and immediately lysed in RIPA buffer (50 mM Tris-HCl, pH 7.4, 150 mM NaCl, 1% (v/v) Nonidet P-40, 0.5% (v/v) sodium deoxycholate and 1 mM EDTA) supplemented with 4 mM *N*-ethylmaleimide. Lysates were immediately boiled for 5 min in the presence of 1% SDS and then diluted 10 times with RIPA buffer. Ubiquitinated AKT was isolated by immunoprecipitation using anti-AKT and detected by IB using an anti-ubiquitin antibody that specifically recognizes K48 ubiquitin chains.

Full size images are presented in Supplementary Figs 8–11.

### Real-time quantitative PCR

RNA was extracted with TRIzol reagent (Sigma) from either mouse T cells or human PBMCs derived from healthy donors or relapsing-remitting MS patients. Specimen collection was approved by the Committee for the Protection of Human Subjects of the University of Texas Health Science Center at Houston, and informed consent was obtained from all subjects. The RNA samples were subjected to quantitative PCR analyses using the SYBR regent (Bio-Rad). The expression of individual genes was calculated by a standard curve method and was normalized to the expression of Actb. The gene-specific primer sets used (all for mouse genes) were as follows: CCR7, 5′- GCACCATGGACCCAGGGAAACC -3′ and 5′- AGTCATCGGTGACCTCATCTTGGCA -3′; KLF2, 5′- TATCTTGCCGTCCTTTGCCA -3′ and 5′- TTTAGGTCCTCATCCGTGCC -3′; CD62L, 5′- AACGATGACGCCTGTCACAA -3′ and 5′- GTTTCCACACATTCTCCACGG -3′; S1pr1, 5′- GGTGTAGACCCAGAGTCCTGC -3′ and 5′- AGAGGCCTCCGAGAAACAGC -3′; AKT1, 5′- AAGAAGGAGGTCATCGTCGC -3′ and 5′- CTTGAGGGCCGTAAGGAAGG -3′; Itga4, 5′- AAGCATCCCTGGCCACTAC -3′ and 5′- GAGCGCCCCAAGAGATGAAG -3′; Itgb1, 5′- AACTTGTTGGTCAGCAACGC -3′ and 5′- AGCCAATCAGCGATCCACAA -3′; Ninj1, 5′- AGTCGGGCACTGAGGAGTAT -3′ and 5′- TACATTGATGGGCCGGTTCC -3′.

### Statistical analysis

For the EAE clinical scores, differences between groups were evaluated by two-way analysis of variance with Bonferroni’s post test. Other statistical analyses were performed by two-tailed unpaired *t*-test using the Prism software. *P* values less than 0.05 were considered significant and the level of significance was indicated as *P<0.05, **P<0.01, ***P<0.001 and ****P<0.0001.

In our animal studies, three to four mice are required for each group based on our calculation to achieve a 2.3-fold change (effect size) in two-tailed *t*-test with 90% power and a significance level of 5%. All statistical tests justified as appropriate and the data meet the assumptions of the tests. The variance is similar between the groups that are being statistically compared.

## Additional information

**How to cite this article:** Yu, J. *et al*. Regulation of T-cell migration by the kinase TBK1 during neuroinflammation. *Nat. Commun.* 6:6074 doi: 10.1038/ncomms7074 (2015).

## Supplementary Material

Supplementary Information Supplementary Figures 1-11
