# Supplementary material for: Regulation of T-cell activation and migration by the kinase TBK1 during neuroinflammation
Source: Nat Commun. 2015 Jan 21;6:6074. doi: 10.1038/ncomms7074 (PMC4302769; doi:10.1038/ncomms7074)
Supplement: Supplementary Information — Supplementary Figures 1-11 [file ncomms7074-s1.pdf]

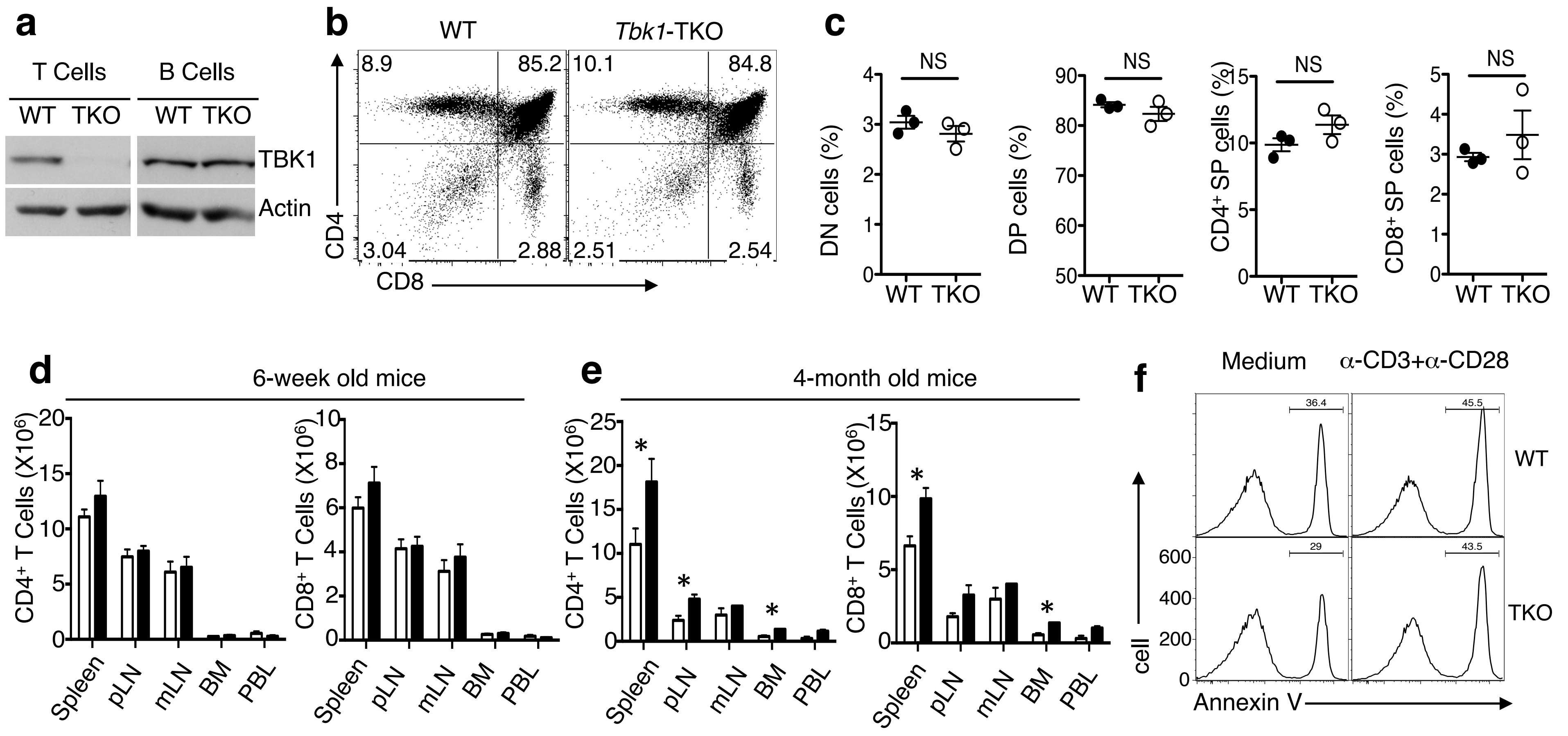

Supplementary Fig. 1. Thymocyte development and peripheral T-cell distribution in WT and *Tbk1*-TKO mice. (**a**) Immunoblot analysis showing the specific ablation of *Tbk1* in T cells, but not in B cells, of *Tbk1*-TKO mice. (**b,c**) Flow cytometric analysis of thymocytes from WT and *Tbk1*-TKO mice (6 wk old). Numbers in quadrants indicate the percentage of $CD4^-CD8^-$ double-negative (DN), $CD4^+CD8^+$ double-positive (DP), $CD4^+$ single-positive (SP), and $CD8^+$ SP thymocytes. Data are representative plot (**b**) and mean ± SD values (**c**) of 3 mice per group. (**d,e**) $CD4^+$ and $CD8^+$ T-cell numbers from spleen, peripheral lymph nodes (pLN), mesenteric lymph nodes (mLN), bone marrow (BM), peripheral blood (PBL) of 6-week old mice (wt=7,ko=6) (**d**) and 8-month old mice (wt=4, ko=3) (**e**). (**f**) Flow cytometry analysis of apoptosis, based on annexin V staining, of WT or *Tbk1*-TKO CD4+ T cells incubated for 48 with medium or anti-CD3 plus anti-CD28. *P<0.05; NS, non-significant.

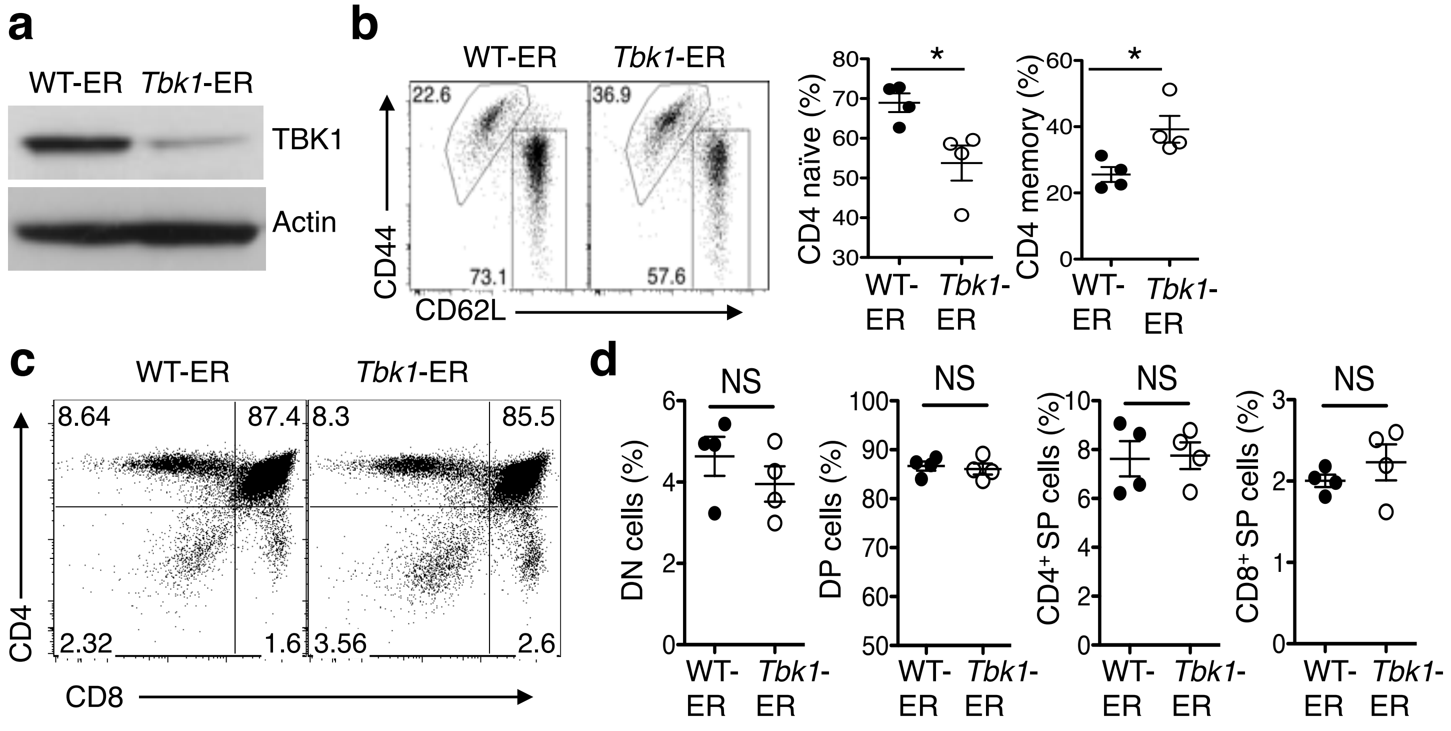

Supplementary Fig. 2. Inducible ablation of TBK1 in adult mice impairs T-cell homeostasis without influencing thymocyte development. (**a**) Immunoblot analysis of TBK1 in T cells isolated from tamoxifen-treated *Tbk1*$^{+/+}$Cre-ER (WT-ER) or *Tbk1*$^{fl/fl}$Cre-ER (*Tbk1*-ER) mice (after two weeks of tamoxifen injection). (**b**) Flow cytometric analysis of the percentage of memory (CD44^{hi}CD62L^{lo}) and naïve (CD44^{lo}CD62L^{hi}) CD4^{+} T cells in the spleen of WT and *Tbk1*-ER mice. Data are representative plot (left) and mean ± SD values (right) of multiple mice (each circle represents a mouse). (**c,d**) Flow cytometric analysis of the thymocyte subpopulations from WT and *Tbk1*-ER mice after weeks after tamoxifen injection. Data are representative plot (**c**) and mean ± SD values (**d**). *P<0.05; NS, non-significant.

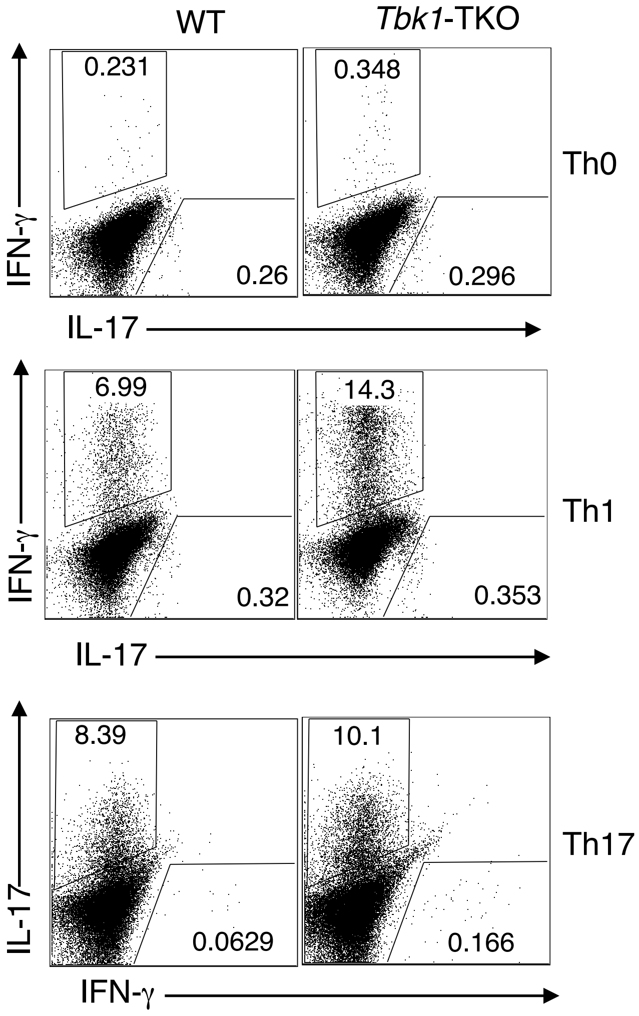

Supplementary Fig. 3. TBK1 deficiency promotes Th1 differentiation *in vitro*. Naive CD4⁺ T cells isolated from WT and *Tbk1*-TKO mice were stimulated for 72 hours with plate-bound anti-CD3 (5μg/ml) and anti-CD28 (1μg/mL) under Th0, Th1, or Th17 conditions followed by flow cytometry to measure the frequency of IFN-γ-producing Th1 cells and IL-17-producing Th17 cells

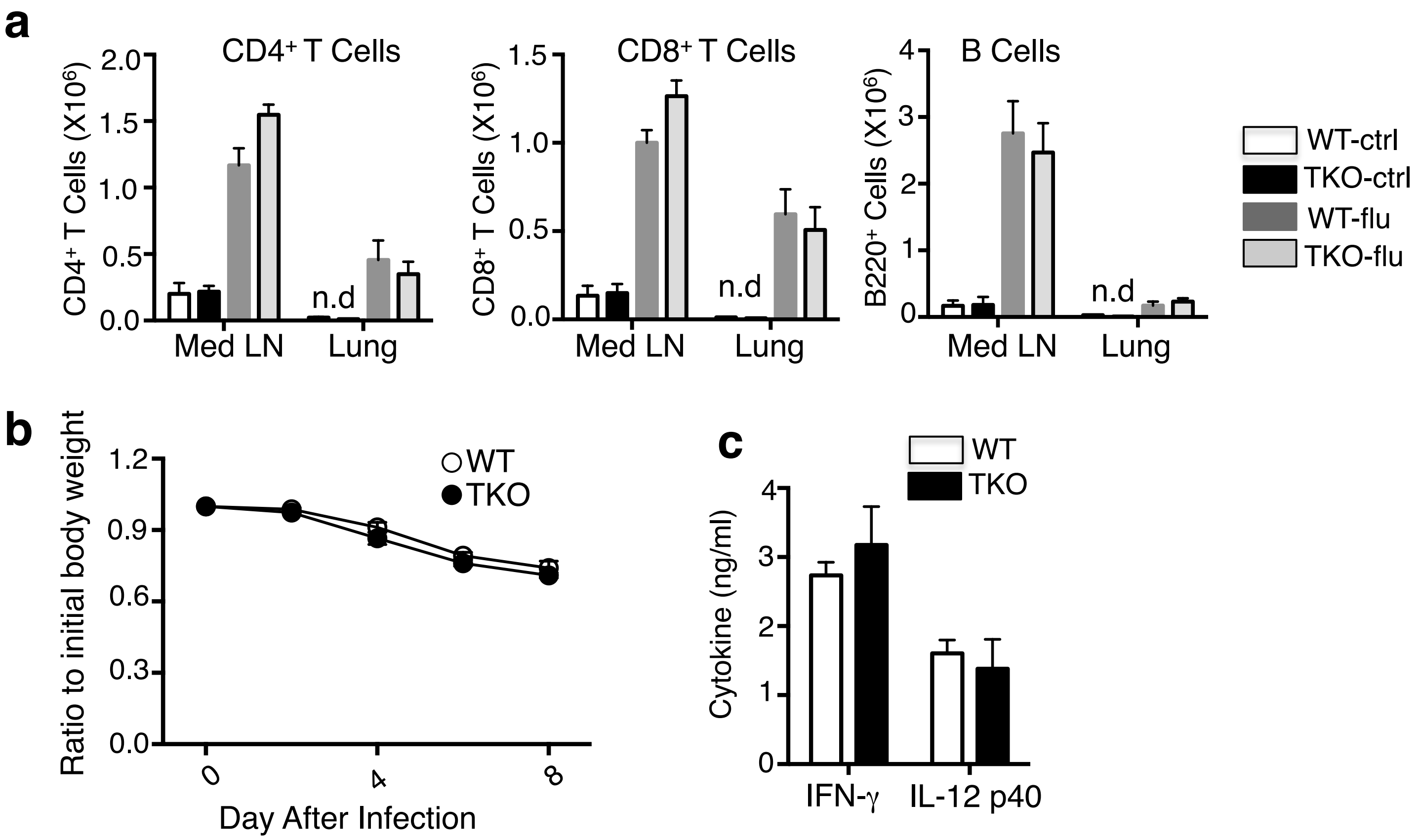

Supplementary Fig. 4. TBK1 deficiency does not affect immune responses against influenza viral infection. Flow cytometry analysis of the indicated cell types in the mediastinal lymph nodes (Med LN) and lung (**a**), bodyweight loss (**b**), and ELISA of cytokine level in the bronchoalveolar lavage fluid (**c**) of WT or *Tbk1*-TKO (TKO) mice infected intranasally with influenza (flu) virus PR8 for 8 days. Data are presented as mean ± s.d. (n=4).

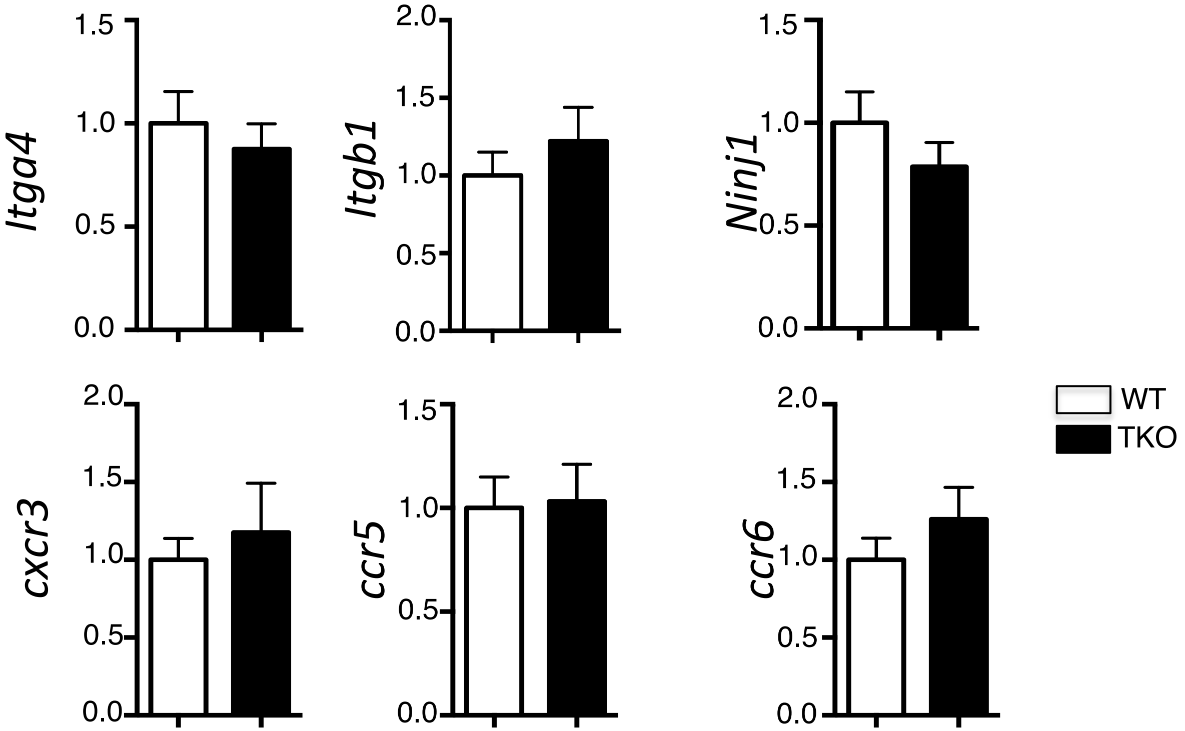

Supplementary Fig. 5. TBK1 is dispensable for the expression of integrin . QPCR analysis of relative gene expression in freshly isolated CD4⁺ T cells from draining LN of WT and *Tbk1*-TKO mice induced with EAE for 18 days.

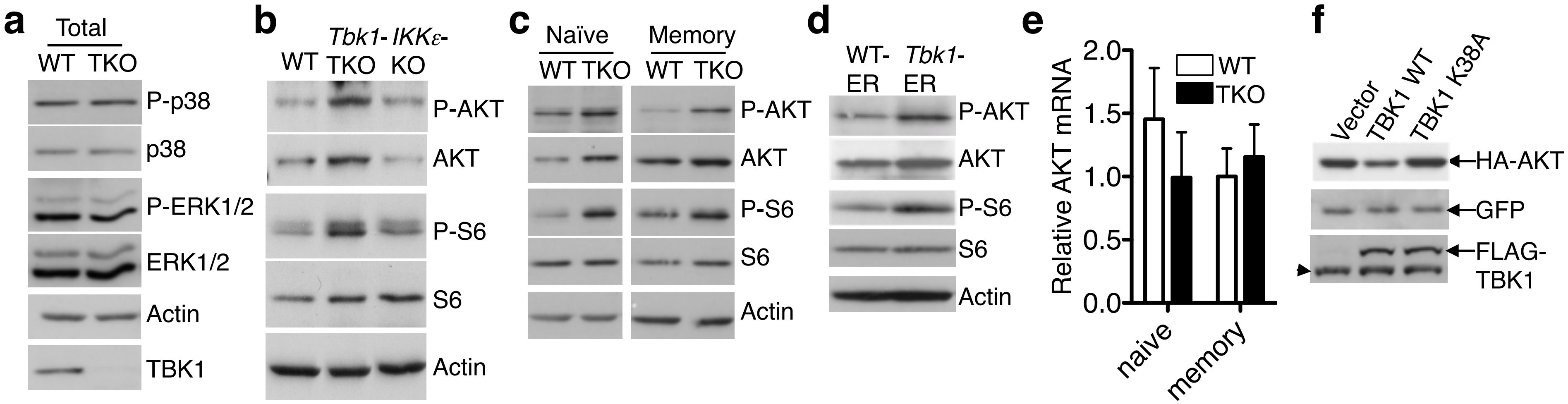

Supplementary Fig. 6. TBK1, but not IKKε, negatively regulates AKT-mTORC1 signaling. (**a-c**) Immunoblot (IB) analysis of the indicated phosphorylated (P-) and total proteins in freshly isolated total CD4$^+$ T cells (**a,b**) or FACS sorted naïve and memory CD4+ T cells (**c**) from WT, *Tbk1*-TKO (TKO), and *IKKε*-KO mice. (**d**) IB analysis using freshly isolated CD4$^+$ T cells of WT-ER and *Tbk1*-ER mice after two weeks of tamoxifen injection. (**e**) QPCR analysis of relative AKT expression in freshly isolated naïve and memory CD4$^+$ T cells from WT and *Tbk1*-TKO mice. (**f**) IB analysis of the indicated proteins using HEK293T cells transfected with an empty vector or the same vector encoding FLAG-tagged TBK1 or its catalytically inactive mutant (K38A), along with vectors encoding GFP and HA-tagged AKT. A non-specific band, detected by the FLAG antibody, was indicated by an arrowhead (bottom panel).

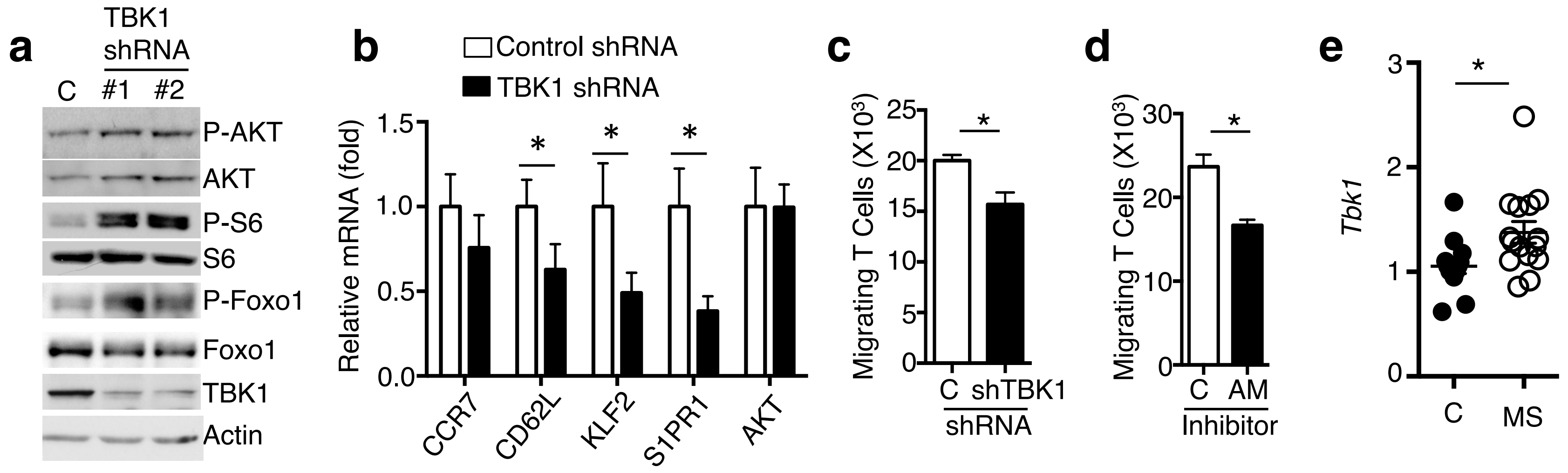

Supplementary Fig. 7. TBK1 regulates AKT-mTORC1 signaling and homing gene expression in human T cells. (**a**) IB analysis of phosphorylated (P-) and total proteins in human PBMCs transduced with a lentiviral vector (pLKO.1) encoding a control luciferase shRNA (C) or two different TBK1 shRNAs. (**b**) QPCR analysis of the indicated genes using human PBMCs transduced with control (C) or TBK1 shRNAs. (**c,d**) In vitro migration assay of activated human $CD4^+$ T cells that were transduced with a control (C) or TBK1-specific shRNA or treated with DMSO (C) and a TBK1 inhibitor, amlexanox (AM, 25 μM). The number of $CD4^+$ T cells migrating through a human brain microvascular endothelial cell monolayer was counted as migrating T cells, and data are representative of three independent experiments performed in duplicates. (**e**) QPCR analysis of *Tbk1* expression in human PBMCs of healthy donors (C, n=13) or MS patients (MS, n=15). *P<0.05.

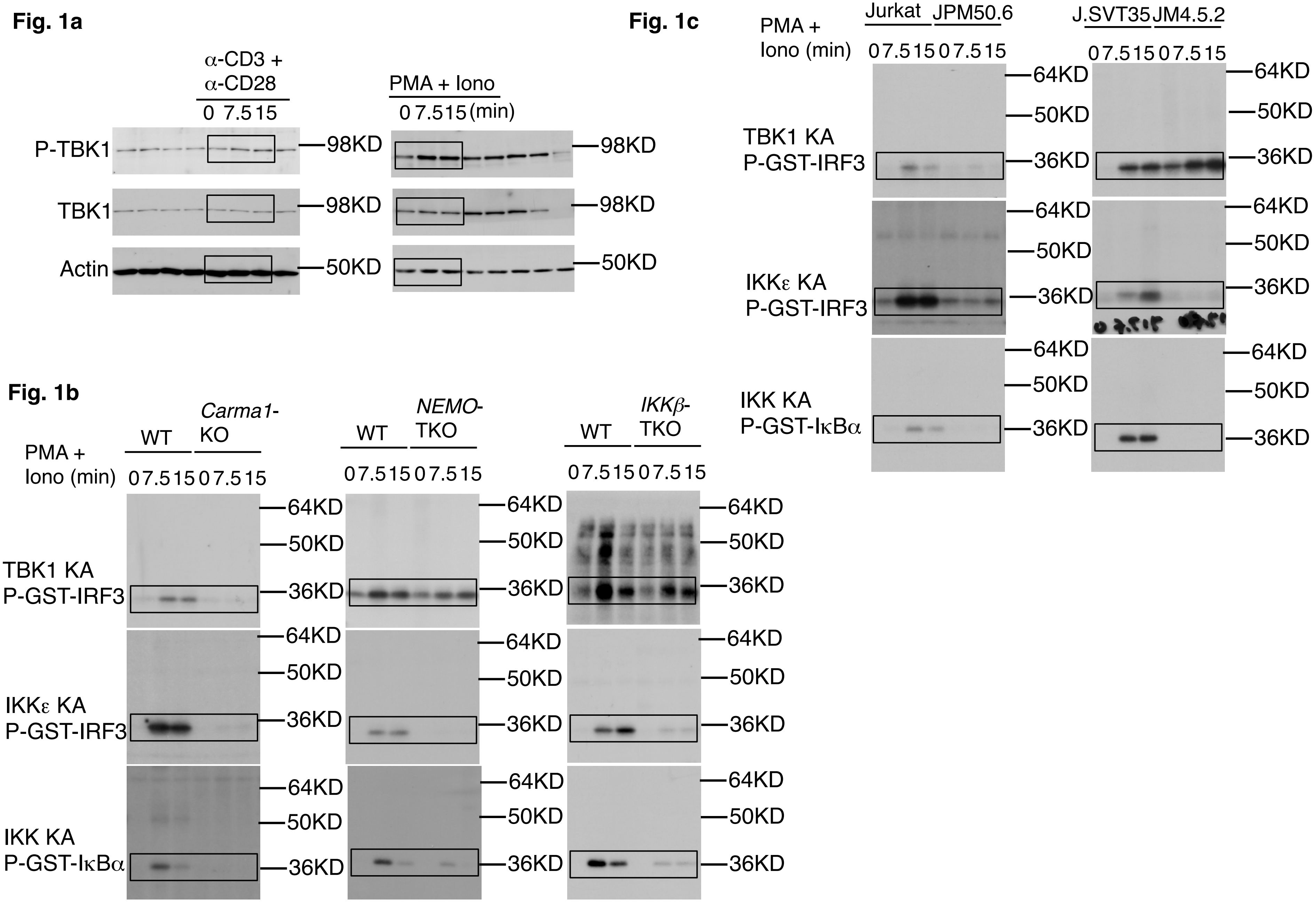

Supplementary Figure 8. Full scans of blots for figures 1a-1c.

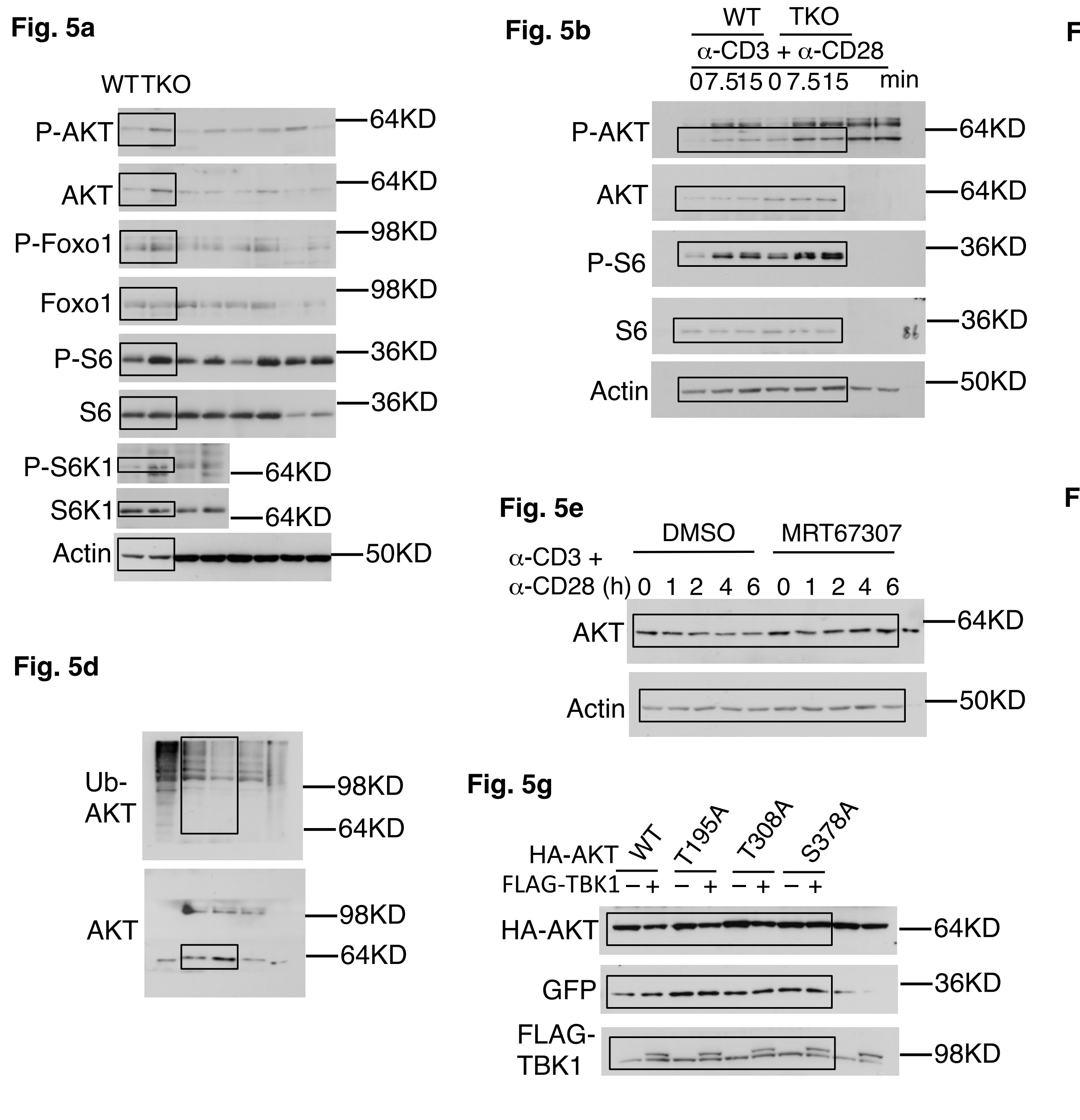

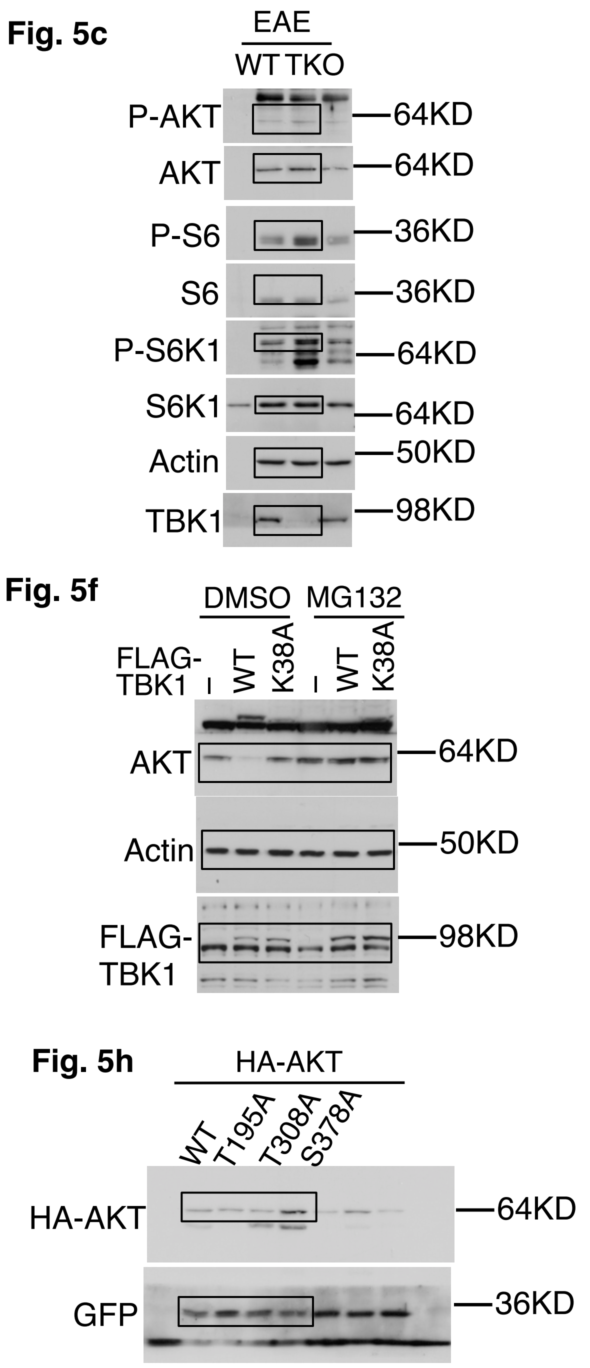

Supplementary Figure 9. Full scans of blots for figures 5a-5h.

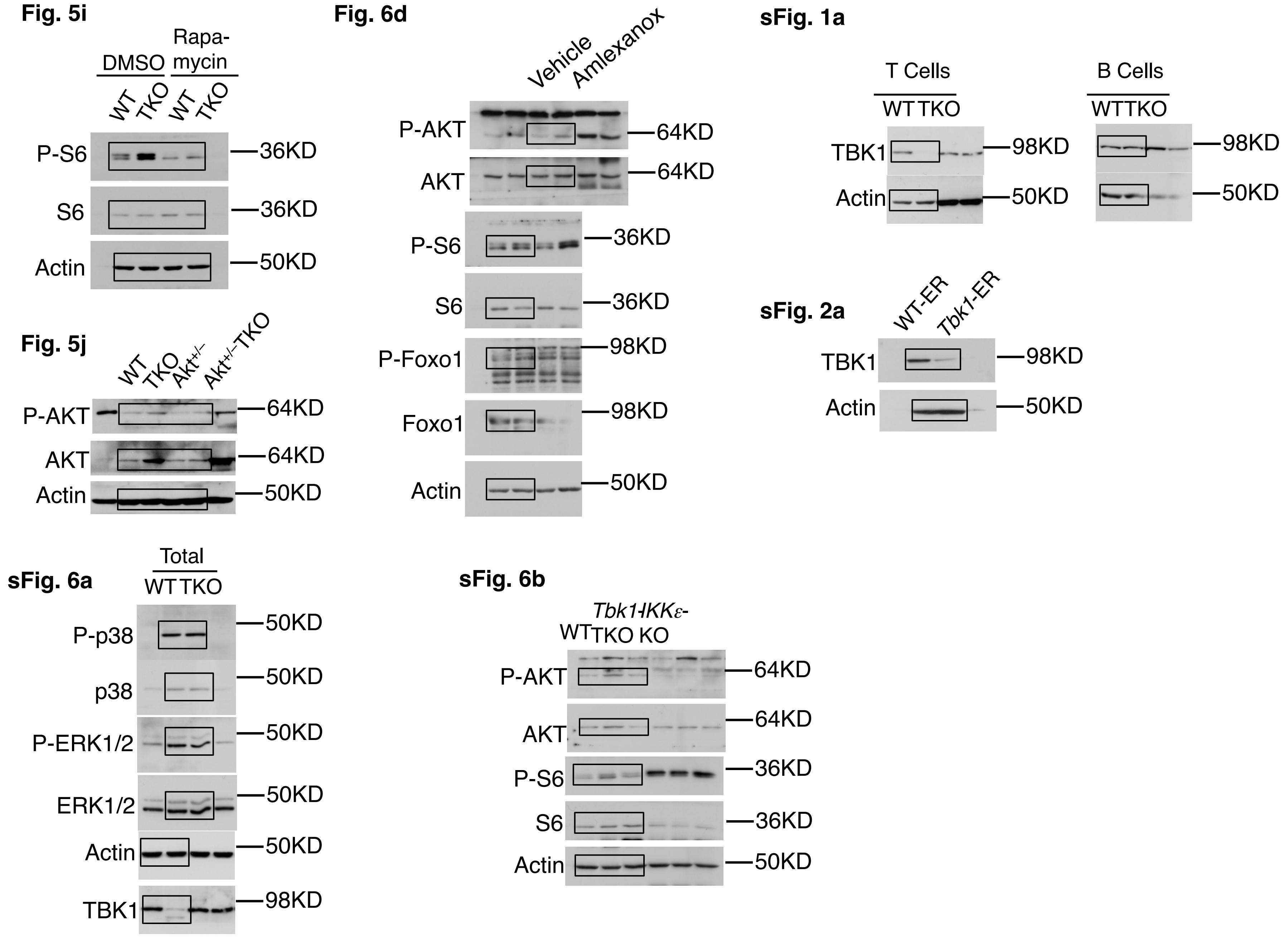

Supplementary Figure 10. Full scans of blots for figures 5i, 5j, 6d, and supplementary figures 1a and 2a.

sFig. 6c

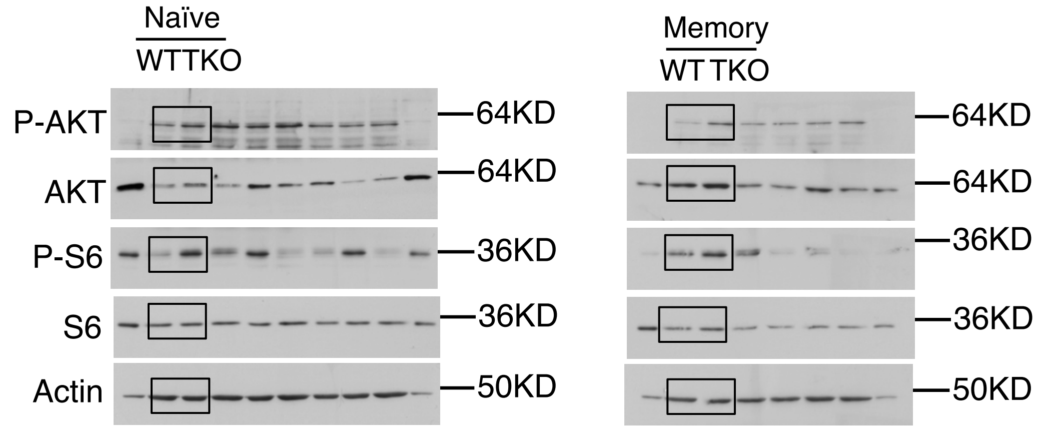

sFig. 6d

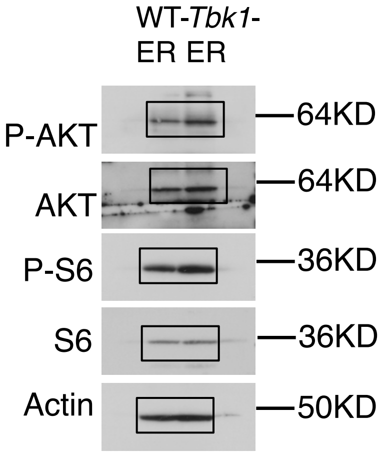

sFig. 6f

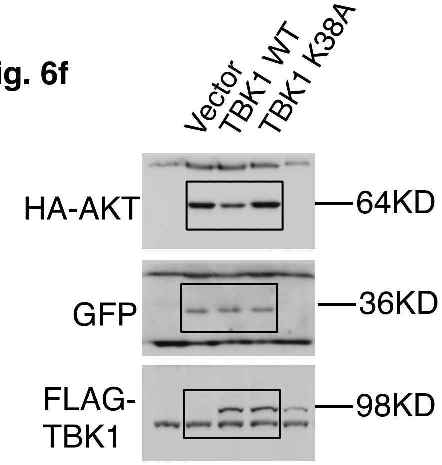

sFig. 7a

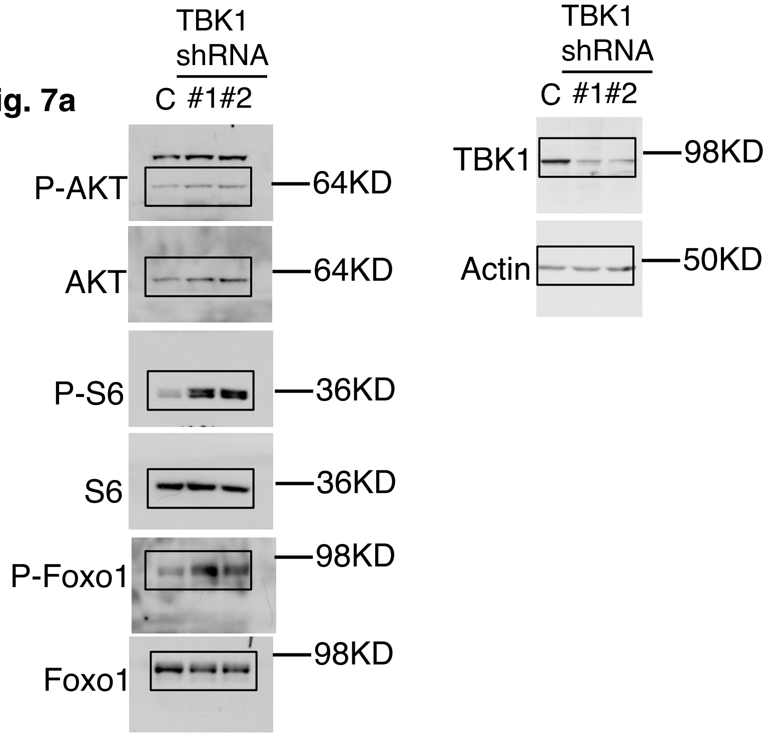

Supplementary Figure11. Full scans of blots for supplementary figures 6c, 6d, 6f, and 7a.
